# Rare variants and survival of patients with idiopathic pulmonary fibrosis: analysis of a multicentre, observational cohort study with independent validation

**DOI:** 10.1016/S2213-2600(25)00045-1

**Published:** 2025-06

**Authors:** Aitana Alonso-González, David Jáspez, José M Lorenzo-Salazar, Shwu-Fan Ma, Emma Strickland, Josyf Mychaleckyj, John S Kim, Yong Huang, Ayodeji Adegunsoye, Justin M Oldham, Iain Stewart, Philip L Molyneaux, Toby M Maher, Louise V Wain, Richard J Allen, R Gisli Jenkins, Jonathan A Kropski, Brian Yaspan, Timothy S Blackwell, David Zhang, Christine Kim Garcia, Fernando J Martinez, Imre Noth, Carlos Flores

**Affiliations:** a Research Unit, Hospital Universitario Nuestra Señora de Candelaria, Instituto de Investigación Sanitaria de Canarias, Santa Cruz de Tenerife, Spain; b Genomics Division, Instituto Tecnológico y de Energías Renovables, Santa Cruz de Tenerife, Spain; c Division of Pulmonary and Critical Care Medicine, University of Virginia, Charlottesville, VA, USA; d Center for Public Health Genomics, University of Virginia, Charlottesville, VA, USA; e Section of Pulmonary and Critical Care Medicine, University of Chicago, Chicago, IL, USA; f Division of Pulmonary and Critical Care Medicine, University of Michigan, Ann Arbor, MI, USA; g Department of Internal Medicine, University of Michigan, Ann Arbor, MI, USA; h National Heart and Lung Institute, Imperial College London, London, UK; i Royal Brompton and Harefield Hospitals, Guy's and St Thomas' NHS Foundation Trust, London, UK; j Division of Pulmonary and Critical Care Medicine, University of Southern California, Los Angeles, CA, USA; k Department of Population Health Sciences, University of Leicester, Leicester, UK; l National Institute for Health Research, Leicester Respiratory Biomedical Research Centre, Glenfield Hospital, Leicester, UK; m Department of Cell and Developmental Biology, Vanderbilt University, Nashville, TN, USA; n Department of Veterans Affairs Medical Center, Nashville, TN, USA; o Division of Pulmonary and Critical Care Medicine, Vanderbilt University, Nashville, TN, USA; p Genentech, San Francisco, CA, USA; q Department of Medicine, Columbia University Irving Medical Center, New York, NY, USA; r and Columbia Precision Medicine Initiative, Columbia University Irving Medical Center, New York, NY, USA; s Weill Cornell Medical Center, New York, NY, USA; t Facultad de Ciencias de la Salud, Universidad Fernando Pessoa Canarias, Las Palmas de Gran Canaria, Spain; u CIBER de Enfermedades Respiratorias (CIBERES), Instituto de Salud Carlos III, Madrid, Spain

## Abstract

**Background:**

Rare pathogenic variants in telomere-related genes are associated with poorer clinical outcomes in idiopathic pulmonary fibrosis (IPF). We aimed to assess whether rare qualifying variants in monogenic adult-onset pulmonary fibrosis genes are associated with IPF survival. Using polygenic risk scores (PRS), we also evaluated the influence of common IPF risk variants in patients carrying the qualifying variants.

**Methods:**

We identified qualifying variants in telomere and non-telomere genes using whole-genome sequences from individuals clinically diagnosed with IPF and enrolled in the Pulmonary Fibrosis Foundation Patient Registry (PFFPR), a large multicentre, observational cohort study (March 29, 2016 to June 15, 2018, n=888). We also derived a PRS for IPF (PRS-IPF) from known common sentinel IPF variants. The primary outcome was the association between qualifying variants and survival. The secondary outcome was the association between qualifying variants and PRS-IPF. We used logistic regression models adjusted for sex, age at diagnosis, and principal components of genetic heterogeneity to examine the mutual relationship of qualifying variants and PRS-IPF. The association between qualifying variants and PRS-IPF with survival was tested using Cox proportional hazard models adjusted for baseline confounders. Validation of the results was sought in data from an independent multicentre, prospective, observational cohort study of IPF in the UK (PROFILE, May 17, 2010 to Sept 5, 2017, n=472), and results were meta-analysed under a fixed-effects model.

**Findings:**

We included 888 patients from PFFPR and 472 from PROFILE, totalling 1360 participants. In the PFFPR, carriers of qualifying variants in monogenic adult-onset pulmonary fibrosis genes were associated with lower PRS-IPF (odds ratio 1·79 [95% CI 1·15–2·81]; p=0·010) and shorter survival (hazard ratio 1·53 [1·12–2·10]; p=7·33 × 10^–3^). Individuals with the lowest PRS-IPF also had worse survival (1·61 [1·25–2·07]; p=1·87 × 10^–4^). These findings were validated in PROFILE and the meta-analysis of the results showed a consistent direction of effect across both cohorts.

**Interpretation:**

We found non-additive effects between qualifying variants and common risk variants in IPF survival, suggesting distinct disease subtypes and raising the possibility of using PRS to guide sequencing prioritisation. Assessing the carrier status for qualifying variants and modelling PRS-IPF promises to further contribute to predicting disease progression among patients with IPF.

**Funding:**

Instituto de Salud Carlos III; Instituto Tecnológico y de Eenergías Renovables; Cabildo Insular de Tenerife; Fundación DISA; National Heart, Lung, and Blood Institute of the US National Institutes of Health; and UK Medical Research Council.

## Introduction

Idiopathic pulmonary fibrosis (IPF) is a rare and progressive disease characterised by lung scarring and poor prognosis, with a median survival of 3–5 years after diagnosis.^1^ Identifying prognostic biomarkers is crucial to improve the clinical management of these patients.

Genetic studies have revealed that both rare and common genetic variants contribute to IPF susceptibility.^2^, ^3^, ^4^, ^5^, ^6^ Although the incorporation of genetic data in IPF diagnosis remains limited, genetic testing is increasingly valuable for predicting disease prognosis.^7^ Genome-wide association studies (GWAS) have revealed multiple common loci involved in IPF progression and survival. For instance, the mucin 5B, oligomeric mucus gel-forming gene (*MUC5B)* risk allele (rs3570950-T), which is the strongest common genetic risk factor known for IPF, is also linked to slower disease progression,^8^ although this association might be subject to an index event bias.^9^ Another genetic risk locus involving the antisense RNA gene of protein kinase N2 (*PKN2*) has been associated with forced vital capacity decline, a common measure for monitoring disease progression.^10^ In addition, the first GWAS of IPF survival found a variant in *PCSK6* associated with differential patient survival.^11^ However, the aggregated effect of all common IPF variants (known as polygenic risk score [PRS]) and its association with patient survival remain undefined.

**Research in context**

**Evidence before this study**

Idiopathic pulmonary fibrosis (IPF) is a devastating disease with a median survival of 3–5 years after diagnosis. The clinical course of the disease varies greatly and the cause of this variation is not well understood. We searched PubMed for studies published in any language from database inception up to Oct 23, 2024, using the terms “idiopathic pulmonary fibrosis”, “rare variants”, “telomere length”, “whole genome sequencing”, “survival “, and “polygenic risk scores”. To date, whole genome and exome sequencing studies have revealed rare deleterious variants within genes involved in two main pathways—telomere maintenance and surfactant metabolism. We identified 54 studies showing that telomere length is associated with poor clinical outcomes, including progressive pulmonary fibrosis, rapid decline of lung function, reduced survival, and poor response to immunosuppression. Among the common genetic variants, the risk allele at rs35705950 of *MUC5B* has been associated with improved survival. Only two studies have reported the association of protein altering variants in telomere genes when stratified by the rs35705950 locus. However, one study considered only *TERT* variants, and the other excluded non-telomere variants from the analysis. In addition, one study examined the effect of combining this risk allele with a monogenic cause on phenotypic characteristics. Although the sample size was small, the study found that this combination had an effect on survival.

To our knowledge, no study has attempted to extensively include rare variants in telomere and non-telomere genes in a survival analysis, nor has any considered the intersected association of rare and common variants in aggregate (as measures of polygenic risk scores).

**Added value of this study**

To the best of our knowledge, this is the first comprehensive study to assess survival outcomes of carriers of both telomere and non-telomere genes. We found a strong association between rare qualifying variants in monogenic pulmonary fibrosis genes and survival in patients from the Pulmonary Fibrosis Foundation Patient Registry and this association was validated in patients from the UK (PROFILE study). Importantly, we observed that non-telomere variants also contribute to this effect. In addition, we found that carriers of rare qualifying variants have low IPF polygenic risk scores, suggesting that rare variants and common variants have non-additive effects in disease progression.

**Implications of all the available evidence**

The clinical heterogeneity of patients with IPF poses a substantial challenge in patient management and decision making. Our findings reinforce the importance of genetic testing in patients with IPF, as rare qualifying variants on both telomere and non-telomere genes are important biomarkers of prognosis. Additionally, our results suggest that these genetic variants have non-additive effects with common IPF risk variants, highlighting the potential role of polygenic risk scores in guiding patient care or the design of clinical trials.

Rare qualifying variants (as a proxy for variants that could have a functional impact on the protein product) in telomere-related genes were associated with poor clinical outcomes among patients with IPF.^5^ However, previous studies have focused mostly on the effects of a few genes (eg, *TERT, RTEL1*, and *PARN*), despite many other telomere and non-telomere genes having been associated with monogenic pulmonary fibrosis.

This evidence supports that multiple genetic factors are involved in distinct mechanisms of IPF pathogenesis and rates of progression. In addition, patients with the *MUC5B* risk allele are less likely to carry rare deleterious variants in adult-onset pulmonary fibrosis genes than non-carriers of the *MUC5B* risk allele, suggesting that the expectation of additive effects of common and rare variants might not be applicable in this case.^5^, ^12^ Nevertheless, the effect of qualifying variants across all known monogenic adult-onset pulmonary fibrosis genes (including telomere and non-telomere genes) in survival and the modifier role of PRS of common risk variants of IPF (PRS-IPF) in qualifying variants carriers remain to be elucidated.

Using whole-genome sequencing from patients with IPF, we determined the prevalence of qualifying variants in monogenic adult-onset pulmonary fibrosis genes and examined the mutual relationships of qualifying variants and PRS-IPF and their association with IPF survival.

## Methods

### Study design and sample description

We assessed the association of qualifying variants and PRS-IPF with survival in patients with IPF. In the discovery stage, we used data from the Pulmonary Fibrosis Foundation Patient Registry (PFFPR), a multicentre, observational cohort study in the USA.^13^ In the second stage, we employed data from the Prospective Observation of Fibrosis in the Lung Clinical Endpoints (PROFILE) cohort for validation,^14^ a multicentre, prospective, observational UK study. We included patients aged 18–85 years with incident IPF and non specific interstitial pneumonia. The primary outcome (time to event) in PFFPR was the time from initial diagnosis to either death or lung transplantation and in PROFILE was the time from diagnosis to death. Both cohorts were right-censored at 60 months reflecting the maximum life expectancy typically observed in patients with IPF. In the current study, the primary outcome was the association between rare qualifying variants and survival. The secondary outcome was the association of qualifying variants and PRS-IPF.

Both studies were conducted according to The Declaration of Helsinki and written informed consent was obtained from all participants. Ethical approval for the PROFILE study was granted by the Royal Free Hospital Research Ethics Committee (London, UK; ethics reference number 10/H0720/12) and University of Northampton Research Ethics Committee (Northampton, UK; ethics reference number 10/H0402/2). Ethical approval for the PFFPR was granted by the Institutional Review Board from the University of Michigan (MI, USA; ethics reference number HUM00111461).

### Procedures

Whole-genome sequencing was performed at Psomagen (Rockville, MD, USA) for PFFPR and Human Longevity (San Diego, CA, USA) for PROFILE. Variant calling was obtained with DRAGEN by aligning to the GRCh38 reference genome. In the PFFPR, telomere length from whole-genome sequencing BAM files (WGS-TL) was estimated using TelSeq 0.0.2.^15^ Individual quality control included identifying quality control outliers, detecting kinship between patients, checking for cross-contamination of samples, and identifying sex discordance between genetically inferred sex and recorded sex.

Qualifying variants were identified in 13 pulmonary fibrosis genes, categorised as either telomere related (*TERC, TERT, TINF2, DKC1, RTEL1, PARN, NAF1*, and *ZCCHC8*) or non-telomere related (*SFTPC, SFTPA1, SFTPA2, SPDL1*, and *KIF15*; appendix pp 4–5, 9). For simplicity, we refer to this gene set as monogenic adult-onset pulmonary fibrosis genes. These variants were annotated as the total set of qualifying variants (appendix pp 10–17). Additionally, qualifying variants were manually classified according to American College of Medical Genetics and Genomics guidelines^16^ as P (pathogenic), LP (likely pathogenic), or VUS (variant of uncertain significance). Variants classified as P or LP comprised the set of pathogenic variants, and ClinVar variants were cross-referenced and annotated as VUS, P, or LP. For sensitivity analysis, we defined six additional categories for qualifying variants. We also assessed a category of rare synonymous variants, expected to capture neutral variation, to use as a null model in the association analyses. These criteria are summarised in the appendix (p 22).

Principal components were calculated after performing genotyping quality control using PLINK v.1.9^17^ (appendix p 5, 35). The PRS-IPF for each patient was derived using the 19 previously published genome-wide significant IPF variants (appendix p 6, 23).^18^ For sensitivity analysis, PRS telomere length (PRS-TL) were estimated based on the 20 common variants that were previously found to be associated with leukocyte telomere length^19^ (appendix p 6, 24).

### Statistical analysis

Descriptive statistics were provided as mean (SD) or median (IQR) and percentages for continuous (quantitative) and categorical (binary) data, respectively. Categorical variables were compared using a Chi squared test or a Fisher's exact test as indicated.

We used Student's *t* test and the Kolmogorov–Smirnov test to compare the mean PRS values and their distributions between qualifying variant carriers and non-carriers. We assessed the relationship between the presence of qualifying variants and the PRS with logistic regression models, adjusted for sex, age at diagnosis, and principal components of genetic heterogeneity. To determine how many principal components to include in the model, we calculated the proportion of genetic variance explained by the first 10 principal components. Since the first two principal components accounted for a significant proportion of the variance (31·29% and 11·09%, respectively), we included only these two in the models (appendix p 35). Multivariable regression models were employed to assess the association of qualifying variants in telomere genes, age, sex, and principal components on WGS-TL.

Cox proportional hazard models were used to estimate hazard ratios (HR) and 95% CIs for the association between qualifying variants, WGS-TL, PRS, and survival. The models were adjusted for sex, age at diagnosis, principal components 1 and 2 for genetic heterogeneity, smoking history, diffusing capacity for carbon monoxide percentage predicted, forced vital capacity percentage predicted, and *MUC5B* risk allele carrier status whenever necessary. To account for unobserved heterogeneity, we also estimate HR and 95% CI using frailty models of survival.

The proportional hazards assumption of each covariate was assessed using Schoenfeld residuals (appendix p 25). When variables did not satisfy the proportional hazards assumption, weighted Cox regression models were used complimentarily to estimate average HR. For visualising survival differences, we generated Kaplan–Meier survival plots and tested the differences using log-rank tests. Results from the PFFPR and PROFILE studies were meta-analysed under a fixed-effects model to assess the directional concordance of associations. Statistical analyses were performed with R version 4.3.1, with p values less than 0·05 considered significant. The Survival R package (version 3.5–7) and the coxphw R package (version 4.0.3) were used for survival analysis and the Meta R package was used for meta-analysis of results.

### Role of the funding source

The funders of the study had no role in study design, data collection, data analysis, data interpretation, or writing of the manuscript.

## Results

**Figure 1:**
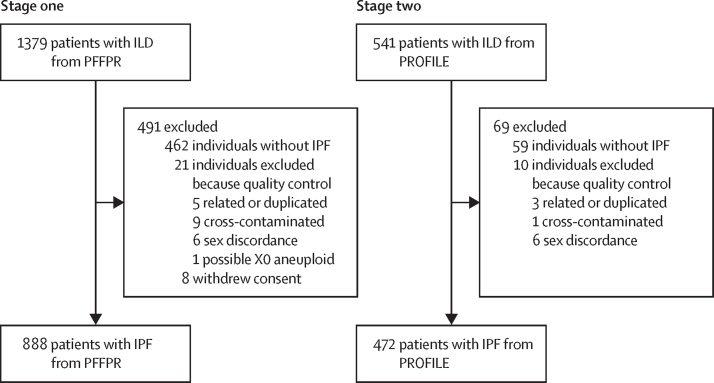
Patient cohorts included from the PFFPR and PROFILE studies ILD=interstitial lung disease. IPF=idiopathic pulmonary fibrosis. PFFPR=Pulmonary Fibrosis Foundation Patient Registry. PROFILE=Prospective Observation of Fibrosis in the Lung Clinical Endpoints. PRS=polygenic risk scores.

Data were collected from March 29, 2016 to Oct 3, 2022 for PFFPR and from May 17, 2010 to Sept 5, 2017 for PROFILE. In the discovery stage, we included 888 patients clinically diagnosed with IPF from the PFFPR from March 29, 2016 to June 15, 2018. 176 (20%) were familial cases. In the second stage, we included 472 clinically diagnosed patients with IPF from the PROFILE cohort, recruited in the UK from May 17, 2010 to Sept 5, 2017, and followed up for 3 years to track disease progression (figure 1). Family history data were not available for this cohort. Ethnicity data were not available for patients from PROFILE. Figure 1 summarises the number of individuals excluded and the reasons for exclusion. For further details, including patient baseline characteristics, see the appendix (pp 4, 8).

The mean age of patients was 71·0 years (SD 7·8) in the PFFPR and 70·7 years (7·9) in PROFILE, and most patients were male (676 [76%] in PFFPR and 366 [78%] in PROFILE). We identified 131 qualifying variants in monogenic adult-onset pulmonary fibrosis genes in 144 patients from the PFFPR resulting in a diagnostic yield of finding a qualifying variant of 16·2% (95% CI 13·8–18·6; table, appendix pp 10–16, 36). We found a strong association between WGS-TL and carrier status of qualifying variants in telomere genes (p=4·21 × 10^–4^) and no association among carriers of qualifying variants in non-telomere genes (appendix pp 28, 37). Carriers of the risk *MUC5B* allele for IPF (rs35705950 TT or TC genotype) had lower prevalence of qualifying variants (84 [13·5%] of 622) compared with people carrying the protective GG genotype (60 [22·6%] of 266; p=1·03 × 10^–3^). See appendix p 7 for further information.

**Table:** Count of qualifying variants in monogenic adult-onset pulmonary fibrosis genes identified in patients from the PFFPR*

	**Number of variants**	**Pathogenic or likely pathogenic**	**Variant of uncertain significance**	**Gene category**
Total variants	131	58	73	..
*KIF15*	15	3	12	Non-telomere
*SPDL1*	9	2	7	Non-telomere
*SFTPC*	6	0	6	Non-telomere
*SFTPA2*	1	0	1	Non-telomere
*SFTPA1*	1	0	1	Non-telomere
*ZCCHC8*	5	1	4	Telomere
*TINF2*	3	3	0	Telomere
*PARN*	16	12	4	Telomere
*RTEL1*	33	18	15	Telomere
*DKC1*	1	0	1	Telomere
*TERC*	4	0	4	Telomere
*NAF1*	6	1	5	Telomere
*TERT*	31	18	13	Telomere

PFFPR=Pulmonary Fibrosis Foundation Patient Registry.

* Filtered by frequency (AF<0·0005) and CADD score (>15).

**Figure 2:**
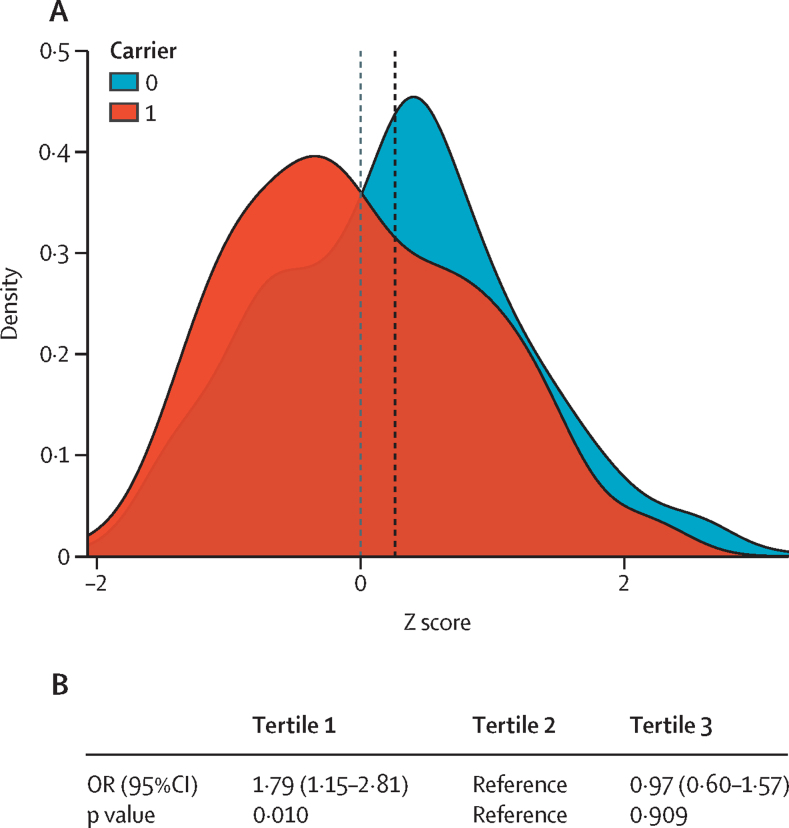
Association between prevalence of qualifying variants and PRS-IPF in the PFFPR (A) Distribution of PRS-IPF in qualifying variant carriers (1) and non-carriers (0). Vertical dotted lines represent the mean value of the distribution. (B) Risk of carrying a qualifying variant for patients with low polygenic risk (tertile 1) and high polygenic risk (tertile 3) compared with those in the middle tertile (tertile 2). The OR and the 95% CIs were estimated using logistic regression adjusted for sex, age of diagnosis, and principal components 1 and 2. IPF=idiopathic pulmonary fibrosis. OR=odds ratio. PRS=polygenic risk scores.

We investigated whether individuals with lower polygenic risk scores were more likely to carry qualifying variants than those with higher polygenic risk scores. We found significant differences in the mean distribution of PRS-IPF between qualifying variant carriers and non-carriers (Student's *t* test: *t*=3·26, p=1·30 × 10^–3^; Kolmogorov–Smirnov test: *D*=0·19, p=3·74 × 10^–4^; figure 2A). When patients were stratified into PRS-IPF tertiles, patients in the lowest tertile (low PRS-IPF) had an increased risk of carrying a qualifying variant (OR 1·79 [95% CI 1·15–2·81], p=0·010; figure 2B) compared with patients in the highest tertile (high PRS-IPF). The association persisted when the cohort was divided into two PRS-IPF categories, low and high (1·74 [1·20–2·53], p=3·57 × 10^–3^; appendix p 38).

Excluding the *MUC5B* locus from the PRS-IPF calculations yielded non-significant differences in the mean and distribution of PRS-IPF between qualifying variant carriers and non-carriers (appendix p 39). Qualifying variants remained numerically higher but non-significantly different in the lowest PRS tertile patients compared with the highest (OR 1·60 [95% CI=1·01–2·54], p=0·05; appendix p 39). No significant associations were found between qualifying variants and PRS-TL (appendix pp 40–42).

**Figure 3:**
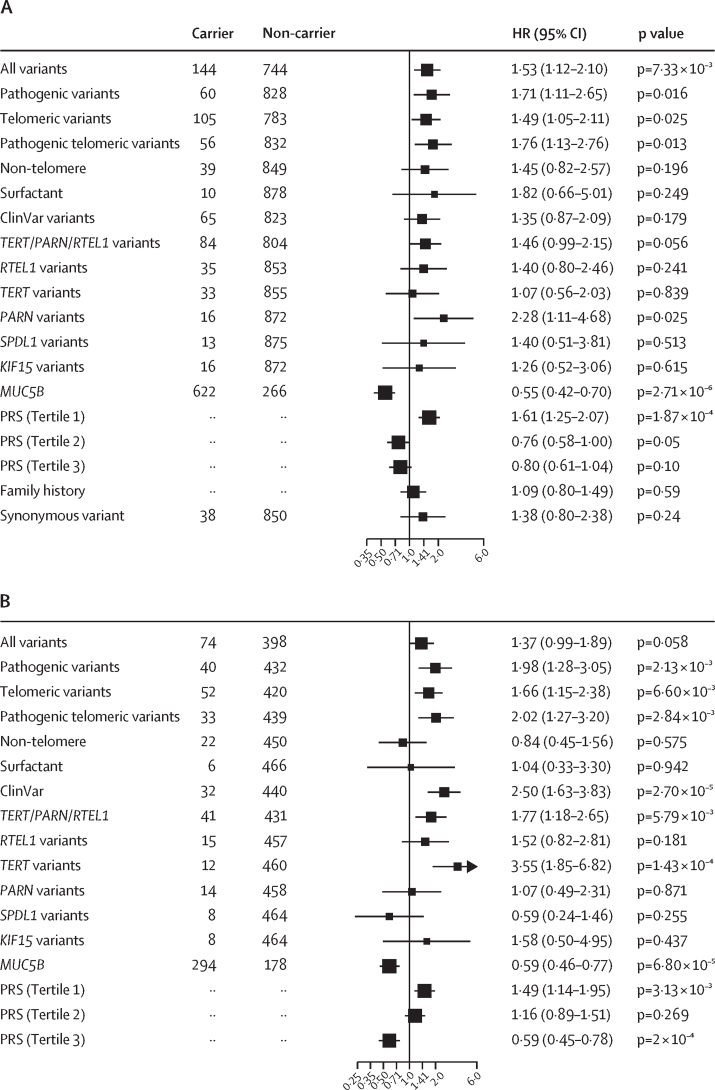
Qualifying variants, *MUC5B* risk allele, PRS-IPF, and family history effect on survival PFFPR (A) and PROFILE (B). All analysis were performed using Cox regression models adjusted for sex, age at diagnosis, principal components 1 and 2, smoking history, forced vital capacity percent predicted, and diffusing capacity for carbon monoxide percent predicted, and the *MUC5B* risk allele whenever necessary. The X-axis shows HR; the black solid line corresponds to the HR=1·0. The boxes correspond to adjusted HR and horizontal lines correspond to 95% CIs. HR=hazard ratio. IPF=idiopathic pulmonary fibrosis. PFFPR=Pulmonary Fibrosis Foundation Patient Registry. PROFILE=Prospective Observation of Fibrosis in the Lung Clinical Endpoints. PRS=polygenic risk scores of IPF.

Qualifying variant carriers were associated with reduced survival (HR 1·53 [95% CI 1·12–2·10], p=7·33 × 10^–3^; log-rank test p=0·022; figure 3A). The same was found when analysis only involved qualifying variants that were classified as P or LP variants (1·71 [1·11–2·65], p=0·016; log-rank test, p=0·043). However, no significant association was found for ClinVar variants alone (1·35 [0·87–2·09], p=0·18; figure 3A, appendix p 43). Further analyses showed that qualifying variants in *PARN* were particularly associated with worse survival (2·28 [1·11–4·68], p=0·025; log-rank test, p=0·035; figure 3A, appendix p 43).

Results were non-significant for the impact of non-telomere and surfactant variants on survival (figure 3A, appendix p 43). The analysis excluding qualifying variant carriers of telomere variants, as they are expected to have worse survival, revealed an increased but still non-significant effect for non-telomere variants (HR 1·64 [95% CI 0·92–2·90], p=0·09) and of surfactant variants (2·07 [0·75–5·75], p=0·16; data not shown), supporting that qualifying variants in these genes also contribute to poorer survival. As an internal control, we found no association and low effect sizes between survival and rare synonymous variants in the same monogenic adult-onset pulmonary fibrosis genes (HR 1·38 [95% CI 0·80–2·38], p=0·24; figure 3A).

We performed additional sensitivity analyses. First, excluding *PARN* qualifying variant carriers attenuated effect size albeit the results remained significant (HR 1·46 [95% CI 1·04–2·03], p=0·027; appendix p 44). Second, we found that family history of pulmonary fibrosis (1·09 [0·80–1·49], p=0·59; figure 3A) and WGS-TL estimates (0·88 [0·74–1·06], p=0·17; data not shown) were not predictive of IPF survival. Third, using alternative and stricter definitions of qualifying variants in the analyses (appendix p 22), we found that the effect had consistent directionality across all qualifying variants definitions (appendix p 45). Finally, random effect models considering explanatory variables for the frailty term confirmed that qualifying variants robustly associated with poorer survival (appendix pp 29–31).

**Figure 4:**
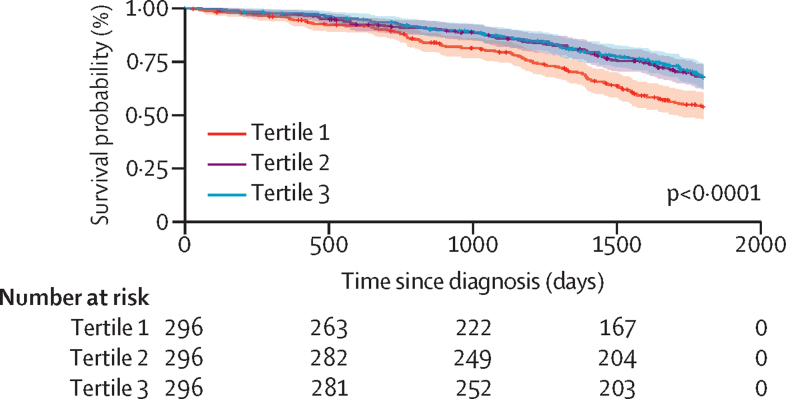
Association between PRS-IPF and survival in the PFFPR Patients with low polygenic risk of IPF (tertile 1) have worse survival than patients with high polygenic risk of IPF (tertile 2 and tertile 3). IPF=idiopathic pulmonary fibrosis. PFFPR=Pulmonary Fibrosis Foundation Patient Registry. PRS=polygenic risk scores.

We then examined whether the polygenic component of IPF was also associated with survival. As PRS-IPF values were mainly influenced by the *MUC5B* effect, we did not adjust for the risk *MUC5B* genotype. We found that the lowest PRS-IPF tertile was associated with the worst survival (log-rank test, p=1·80 × 10^–4^; HR 1·61 [95% CI 1·25–2·07], p=1·87 × 10^–4^; figure 3A, figure 4). PRS-TL was not associated with survival (appendix pp 46–47).

A sensitivity analysis excluding the *MUC5B* locus from the PRS-IPF calculation yielded non-significant results (appendix p 48). Additionally, we stratified patients by qualifying variant carrier status and assessed the effect of PRS-IPF in each group. The analyses showed that patients with lower PRS-IPF were associated with worse survival in both groups, although this association was attenuated among carriers (HR 1·76 [95% CI 0·99–3·15], p=0·055) compared with non-carriers (1·54 [1·16–2·04], p=2·53 × 10^–3^; appendix p 49). Similar results were obtained when we assessed the effect of the *MUC5B* rs35705950 genotypes on survival among both groups (appendix p 49). We also defined subtypes of patients combining information of PRS-IPF and carrier status of qualifying variants and compared their survival against the non-carriers with high PRS-IPF as the reference group. We found that carriers with high-risk PRS-IPF did not have significantly worse survival (HR 1·66 [95% CI 0·96–2·86], p=0·068; appendix p 49).

For patients in PROFILE, there were no statistical differences in the mean and the distribution of PRS-IPF between qualifying variant carriers and non-carriers (Student's *t* test: *t*=1·38, p=0·17; Kolmogorov–Smirnov test: *D*=0·13, p=0·24). No statistically significant difference of qualifying variants in the patients with a lower polygenic component of IPF was observed compared with patients with a higher polygenic component (OR 1·29 [95% CI 0·78–2·15], p=0·31; appendix p 50).

The association between pathogenic and telomeric variants and IPF survival was validated in PROFILE (figure 3B, appendix p 51). These results were preserved adding a simple random effect term to the Cox model or using weighted Cox regression models (appendix pp 29–32).

Since the primary outcome of time to event differed between the two cohorts, we performed a sensitivity analysis to determine its effect in the results. As the timing of referral for lung transplantation is uncertain, we ran the survival analyses at different follow-up intervals (24, 36, and 48 months) in PROFILE and observed consistent effect sizes for the main findings irrespective of the intervals (appendix p 33). In addition, survival analyses in the PFFPR cohort after excluding patients who had undergone lung transplant did not alter the results (appendix p 34).

**Figure 5:**
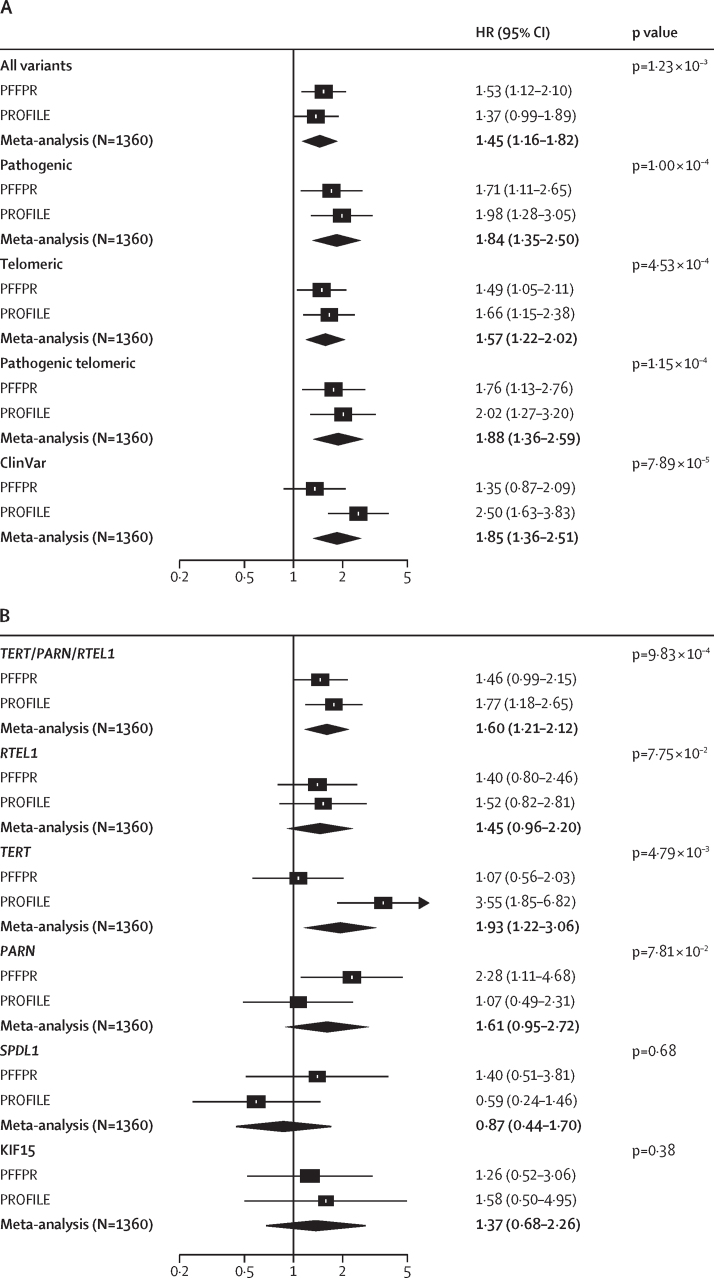

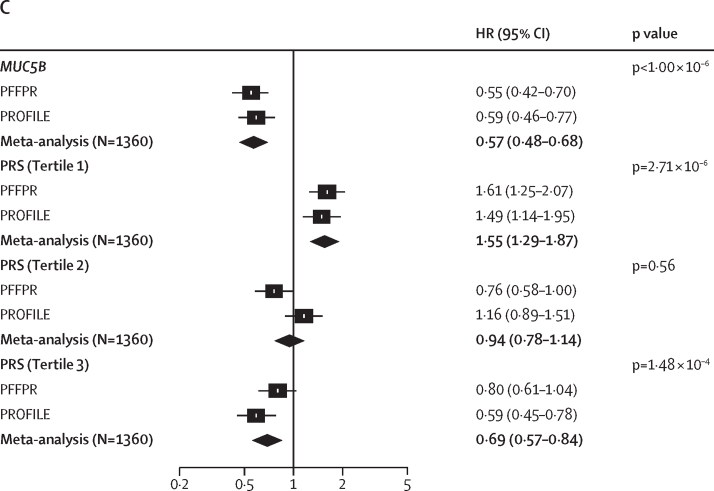
Meta-analysed results from adjusted PFFPR and PROFILE Cox regression models (A) HRs by variant category. (B) HRs by gene. (C) HRs by PRS-IPF and *MUC5B*. HR=hazard ratio. IPF=idiopathic pulmonary fibrosis. PFFPR=Pulmonary Fibrosis Foundation Patient Registry. PROFILE=Prospective Observation of Fibrosis in the Lung Clinical Endpoints. PRS=polygenic risk scores of idiopathic pulmonary fibrosis.

The meta-analysis of results from the PFFPR and PROFILE cohorts showed a consistent direction of effect across all categories and supported a robust association between qualifying variants (including “all variants”, “pathogenic”, “telomeric”, and “pathogenic telomeric”) and PRS-IPF tertiles with survival (figure 5).

## Discussion

This study shows that patients with IPF carrying qualifying variants in monogenic adult-onset pulmonary fibrosis genes are at an increased risk of reduced survival compared with non-carriers. Additionally, we found that qualifying variant carriers tend to exhibit a lower polygenic risk component for IPF suggesting non-additive effects between rare and common genetic variants. This result indicates that the interplay between these rare and common variants define distinct genetic subtypes of IPF. These key findings were replicated across two independent studies including 1360 patients.

The association between telomere variants and worse IPF outcomes is well established. Carriers of qualifying variants in *TERT, PARN, TERC*, or *RTEL1* tend to have earlier disease onset, more rapid lung function decline, and poorer survival compared with non-carriers,^5^, ^12^, ^20^ findings that are mirrored in individuals with short telomere length.^5^, ^21^ However, the exact correlation between rare telomere-related variants and telomere length remains unclear, and the effect sizes of known common variants on telomere length are too small to fully explain this relationship.^5^ In the PFFPR, WGS-TL estimates showed a strong correlation with qualifying variants in telomere genes, providing evidence of their functional impact. However, WGS-TL estimates were not predictive of IPF survival. Although short telomere length has been consistently described as a prognostic biomarker in pulmonary fibrosis cohorts, its value in IPF-specific cohorts remains less clear as they are enriched for shorter telomere length. As a result, the effect of telomere length on survival is smaller compared with other pulmonary fibrosis subtypes, and its effect on survival might not be accounted for until a critical threshold of telomere length is reached (eg, telomere length <1st percentile).^22^ Moreover, the reliability of WGS-TL estimates might be limited by sequencing artefacts, as some studies have reported weaker associations when compared with direct telomere length measurement methods, such as quantitative PCR.^5^ Finally, it should be noted that the influence of non-telomere-related factors on survival cannot be captured by these estimates. Our study stands out by assessing the aggregate effect of qualifying variants across both telomere and non-telomere genes on IPF survival. Although the effect sizes were smaller than for models that included only telomere-related variants, the most robust associations were found when considering all qualifying variants across telomere and non-telomere genes. However, non-telomere variants showed no significant difference in survival when carriers of telomere variants were excluded from the analysis.

Identifying prognostic markers in pulmonary fibrosis is crucial to improve clinical management of the condition. As antifibrotic drugs can only slow disease progression,^23^, ^24^ early identification of patients with poorer prognosis could guide decisions on more aggressive treatments, such as lung transplantation. This information could also enhance the efficiency of clinical trials.^25^ Previous studies support that a family history of pulmonary fibrosis is associated with worse patient survival.^26^ However, familial pulmonary fibrosis could encompass a broad group of patients with interstitial lung disease (ILD) whose disease progression is dependent on different factors. For instance, one factor will be considering the risk linked to rare telomere-related variants,^27^ to common genetic variants,^28^ and to short age-adjusted telomere length,^29^ since they are interrelated with the family history of pulmonary fibrosis. Our findings revealed non-additive opposite effects of qualifying variants and PRS-IPF on survival. In contrast with the results reported by Zhang and colleagues,^5^ we found no apparent association with PRS-TL, suggesting that genetically predicted telomere length might not be meaningful for IPF populations. This result is in line with the WGS-TL findings, which were also not predictive of IPF survival. Finally, we did not find an association between family history and IPF survival, although these results should be interpreted with caution considering that self-reported family history is prone to recall bias.

By contrast, our results show that qualifying variants are robust predictors of prognosis in patients with IPF. Despite this, the use of genetic testing in IPF is not yet a generalised practice and current guidelines only recommend its use in familial forms of pulmonary fibrosis.^7^, ^30^ Nonetheless, we and others show the existence of a substantial burden of qualifying variants in cases in which familial history is not confirmed. It is therefore an urgent necessity to define additional criteria to improve the diagnostic yield of genetic tests in these patients. On one side, the term familial might need to be re-evaluated to better identify hereditary cases. Although IPF is the most common subtype of ILD among familial cases, discordant diagnoses can occur within relatives carrying the same pathogenic variant.^20^ Recent studies have reported rare telomere variants in other ILD subtypes, such as hypersensitivity pneumonitis^31^ or rheumatoid arthritis-associated ILD,^32^ which typically have a better prognosis than IPF. Consequently, these cases might be more easily overlooked within families. To address this, it is essential to take a thorough family history in IPF cases, considering this clinical heterogeneity. Additionally, for some complex diseases, PRS have been suggested as a promising strategy for prioritising patients who should undergo genetic sequencing.^33^ For example, in prostate cancer, the high penetrant variant *HOXB13 G84E* is most common in cases with the lowest PRS.^34^ The success of this approach depends on the strength of the PRS and the genetic architecture of the disease.^33^ IPF meets several criteria that make it suitable for applying PRS for patient prioritisation since there is modest genetic heterogeneity in this disease and the number of susceptibility genes appears to be markedly less than other complex diseases;^18^ the cause of IPF is driven by both rare, highly penetrant variants that explain monogenic-like presentations, and common variants with low effect sizes that contribute for a polygenic disorder;^6^ and the PRS-IPF accurately identifies individuals at high risk of interstitial lung abnormalities and IPF.^35^ In agreement, we have found that PRS-IPF values are inversely associated with the likelihood that qualifying variants are present in the patient. This evidence supports the idea that PRS could serve as a valuable tool for prioritising those patients who should undergo a deeper sequence-based analysis.

This study has some limitations. First, we are aware that other genes not considered for the study are also involved in IPF risk. Our analyses were restricted to a limited number of genes showing dominant inheritance and presumably high penetrance. This resulted in the exclusion of well-defined monogenic pulmonary fibrosis genes, such as *ABCA3* or *NOP10,* which show recessive inheritance. In addition, there are no co-segregation data, direct telomere length measures, or functional evidence to accurately classify most qualifying variants as P or LP despite the fact we found correlation between the WGS-TL estimates and qualifying variant carriers in telomere genes. Therefore, we recognise that some variants categorised as qualifying variants might be VUS or benign. To address this limitation, we have provided alternative qualifying variant definitions based on different in silico predictors and allele frequency cutoffs, which consistently showed the same direction of effect in the survival analysis. Finally, we did not account for extrapulmonary manifestations or the phenomenon of genetic anticipation in our models. All these limitations might translate to additional variability that is not fully explained by our main model. However, the use of a random effects model preserved the main findings, suggesting that our results might be transferable to other heterogeneous cohorts.

For PRS analyses, we relied on simple models based on sentinel variants from existing GWAS. For IPF, this implies that the PRS-IPF results are mainly driven by the effect of *MUC5B*. However, a recent study provided support that a common genetic variant score complements the *MUC5B* variant in accurately identifying individuals at high risk of interstitial lung abnormalities and IPF.^35^ Therefore, the use of whole-genome PRS models might have benefits by offering robust associations across cohorts. However, most IPF GWAS conducted to date mainly involve participants of European genetic ancestry. As a result, the predictive power of genome-wide PRS is expected to incompletely transfer to non-European populations, which would limit their generalisability. The fact that the PRS-IPF model is mainly driven by *MUC5B* might represent an advantage. *MUC5B* is one of the most impactful disease-associated common variants in humans, and its association with IPF has been consistently observed across multiple ancestry groups.^36^ As cross-ancestry genetic prediction methodologies are improved and more multi-ancestry GWAS studies are developed in IPF,^4^ genotyping the *MUC5B* IPF risk variant could serve as a simple proxy for further genetic testing decisions.

Finally, despite the main findings being consistent across the PFFPR and PROFILE cohorts, we acknowledge that there were differences in the definition of the primary endpoint. To address this limitation, we conducted sensitivity analyses confirming that the primary findings were not affected, meaning that the results are likely to be generalisable across studies. Additionally, patient recruitment in PROFILE started before the approval of pirfenidone and nintedanib, which might explain the reduced median survival of patients in this cohort compared with the PFFPR. There are also issues related to the genetic background and patients' characterisation in PROFILE. For example, family history was not recorded, making it challenging to determine the enrichment of familial versus sporadic cases. In addition, the prevalence of the *MUC5B* IPF risk allele differs significantly with that of the PFFPR. This difference, coupled with the reduced sample size of PROFILE, might explain why we did not observe significant differences in the mean PRS-IPF values among qualifying variant carriers and non-carriers, despite there being a trend towards enrichment of qualifying variants in patients with lower PRS-IPF.

In conclusion, we found a robust association of telomere and non-telomere gene qualifying variants with IPF patient survival, highlighting the potential role of qualifying variants as biomarkers of prognosis in clinical care. We also show that those qualifying variants were non-additive to common IPF risk variants on survival raising the possibility of using PRS to guide sequencing prioritisation. This study highlights the potential significance of identifying qualifying variants in telomere and non-telomere genes linked to monogenic forms of pulmonary fibrosis in clinical practice.

### Contributors

### Data sharing

Data supporting the findings are available as part of the manuscript or from the supplementary files. Access to the raw whole-genome sequence dataset is restricted to qualified researchers under an agreement with the PFFPR and PROFILE steering committees to protect the privacy of the participants. For further information and to apply for access to the data from the PFFPR before public deposit in BioLINCC, please contact the chair of the ancillary committee (IN) or any other member of the steering committee (https://www.pulmonaryfibrosis.org/pff-registry/pff-patient-registry). For further information and to apply for access to the PROFILE data, please contact admin.mtwc@imperial.ac.uk. Data access requests must be reviewed before release.

## Declaration of interests

CKG has stock or stock options in Rejuvenation Technologies. TSB reports personal fees from Boehringer Ingelheim and grant support from the Department of Veterans Affairs. AA has received consultancy fees from Genentech, Inogen, Medscape, AbbVie, PatientMpower, Brainomix, PureTech, GossamerBio, and Boehringer Ingelheim. IS has received grants from Rayne Foundation and has participated as Data Safety Monitoring or Advisory Board in patientMpower. CF reports receiving consulting, lecture, or educational fees from Fundación Instituto Roche. DZ has received consultancy fees from Boehringer Ingelheim. TMM has received consultancy or speaker's fees from Boehringer Ingelheim, Roche/Genentech, Abbie, Amgen, Astra Zeneca, Bayer, Bridge bio, Bristol-Myers Squibb, CSL Behring, Galapagos, Galecto, GlaxoSmithKline, IQVIA, Merck, Pfizer, Pliant, PureTech, Sanofi, Trevi, Vicore, has participated as Data Safety Monitoring or Advisory Board in Fibrogen, United Therapeutics, and Nerre and has stock options in Qureight. IN has received a grant from Veracyte and consulting fees from Boehringer Ingelheim and Sanofi. JMO has received consulting fees from Boehringer Ingelheim, Lupin pharmaceuticals, AmMax Bio, Roche, and Veracyte, is holder of an issued patent for the TOLLIP TT genotype for NAC use in IPF, has stock options in Gatehouse Bio, and has participated as Data Safety Monitoring Board or Advisory Board for Endeavor Biomedicines, Novartis, and Genentech. LVW reports funding from, consultancy for, or collaboration with GlaxoSmithKline, Genentech, Orion Pharma, Boehringer Ingelheim, AstraZeneca, Nordic Bioscience, Sysmex (OGT), and Galapagos. BY is a current employee of Genentech/Roche with stock options in Roche. RGJ has also served as a consultant to Bristol Myers Squibb, Chiesi, Resolution Therapeutics, AbbVie, AdAlta, Brainnomix, CohBar, Galecto, Glaxo Myers Squibb, RedX, Syndax, and Pliant, has received payment or honoraria for lectures, presentations, speaker bureaus, manuscript writing, or educational events from Boehringer Ingelheim, Chiesi, Roche, PatientMPower, and AstraZeneca, has participated on a data safety monitoring board or advisory board for Boehringer Ingelheim, Galapagos, and Vicore, and has an unpaid role in an advisory board at NuMedii. RGJ is also a trustee of Action for Pulmonary Fibrosis. PLM has received support from AstraZeneca, GlaxoSmithKline, Asthma & Lung UK, and Action for Pulmonary Fibrosis, has received consulting fees from Roche, Boehringer Ingelheim, AstraZeneca, Trevi, Qureight, and Endeavour, has received payment or honoraria for lectures, presentations, speaker bureaus, manuscript writing, or educational events from Boehringer Ingelheim and Roche, and has participated on a data safety monitoring board or advisory board for United Therapeutics. JAK has received funding from the US Department of Defense, Department of Veterans Affairs, Boehringer Ingelheim, Bristol Myers Squibb, and has stock options in APIE. FJM has received funding from Afferent/Merck, Boehringer Ingelheim, Biogen, DevPro, GlaxoSmithKline, Nitto, Patara/Respivant, Roche, Vicore, has received payment or honoraria for lectures, meetings or travel, presentations, speaker bureaus, manuscript writing, or educational events from Boehringer Ingelheim and Chiesi, has participated on a data safety monitoring board or advisory board for Endeavor Biomedicines, and has received equipment, materials, drugs, medical writing, gifts, or other services from Boehringer Ingelheim and Roche. All other authors declare no competing interests.
