# Supplementary material for: Rare variants and survival of patients with idiopathic pulmonary fibrosis: analysis of a multicentre, observational cohort study with independent validation
Source: Lancet Respir Med. 2025 Jun;13(6):495–504. doi: 10.1016/S2213-2600(25)00045-1 (PMC12117017; doi:10.1016/S2213-2600(25)00045-1)
Supplement: Supplementary appendix [file mmc1.pdf]

THE LANCET
Respiratory Medicine

Supplementary appendix

This appendix formed part of the original submission and has been peer reviewed. We post it as supplied by the authors.

Supplement to: Alonso-González A, Jáspez D, Lorenzo-Salazar JM, et al. Rare variants and survival of patients with idiopathic pulmonary fibrosis: analysis of a multicentre, observational cohort study with independent validation. *Lancet Respir Med* 2025; published online April 28. https://doi.org/10.1016/S2213-2600(25)00045-1.

Supplementary material

Rare variants and survival of patients with idiopathic pulmonary fibrosis: analysis of a multicentre, observational cohort study with independent validation

Aitana Alonso-Gonzalez, PhD[1], David Jáspez, MSc[2], José M. Lorenzo-Salazar, MSc[2], Shwu-Fan Ma, PhD[3], Emma Strickland, BA[3], Prof Josyf Mychaleckyj, PhD[4], John S. Kim, MD[3], Yong Huang, PhD[3], Ayodeji Adegunsoye, MD[5], Justin M. Oldham, MD[6], Iain Stewart[7], Prof Philip L. Molyneaux, PhD[7,8], Prof Toby M. Maher, PhD[7,9], Prof Louise V. Wain, PhD[10,11], Richard J. Allen, PhD[10,11], Prof R. Gisli Jenkins, PhD[7], Jonathan A. Kropski, MD[12,13,14], Brian Yaspan, PhD[15], Timothy S. Blackwell, MD[16], David Zhang, MD[17], Christine Kim Garcia, MD[17,18], Fernando J. Martinez, MD[19], Imre Noth, MD[3], and Carlos Flores, PhD[1,2,20,21*]

Supplementary methods **4**
Description of study cohorts 4
Supplementary sequencing and bioinformatics analysis methods 4
Identification of qualifying variants (QVs) in monogenic adult-onset PF genes 5
Principal components of genetic heterogeneity in the cohorts 5
Estimation of polygenic risk scores (PRS) 6
Supplementary results **7**
Prevalence of QVs in the PFFPR 7
Prevalence of QVs in PROFILE 7
Supplementary Tables **8**
Supplementary Table 1. Baseline characteristics and outcomes of IPF patients from stage one and stage two cohorts. 8
Supplementary Table 2. Regions of interest (ROIs) for qualifying variants annotations in hg38. 9
Supplementary Table 3. Qualifying variants identified in the PFFPR 10
Supplementary Table 4. Qualifying variants identified in PROFILE 17
Supplementary Table 5. Alternative definitions for qualifying variants and the rare synonymous used for sensitivity analyses. 22
Supplementary Table 6. Common IPF risk variants and effects considered for PRS-IPF estimation 23
Supplementary Table 7. Common telomere length variants and effects considered for PRS-TL estimation 24
Supplementary Table 8. Schoenfeld residuals test for proportionality assumption of the Cox models. 25
Supplementary Table 9. Genomic predictors of whole-genome sequencing telomere length (WGS-TL) in multivariate linear regression model stratified by groups of carriers with qualifying variants.. 28
Supplementary Table 10. Results of the random effects models with the age at diagnosis as the frailty term. 29
Supplementary Table 11. Results of the random effects models with the predicted FVC as the frailty term. 30
Supplementary Table 12. Results of the random effects models with the predicted DLCO as the frailty term. 31
Supplementary Table 13. Weighted Cox regression analysis in patients carrying rare qualifying variants in PROFILE.. 32
Supplementary Table 14. Cox regression analysis at 24, 36, 48 and 60 months (m) from diagnosis in patients carrying rare qualifying variants in PROFILE. 33
Supplementary Table 15. Cox regression analysis at 60 months (m) from diagnosis in non-transplant patients carrying rare qualifying variants in PFFPR. 34
Supplementary Figures **35**
Supplementary Figure 1. Principal component analysis. 35
Supplementary Figure 2. Distribution of qualifying variants (QV) in monogenic adult-onset pulmonary fibrosis (PF) genes in the PFFPF and PROFILE cohorts. 36
Supplementary Figure 3. Violin plots comparing telomere length estimated by whole-genome sequencing (WGS-TL) between carriers of qualifying variants in telomere genes and non-carriers. 37
Supplementary Figure 4. Association between prevalence of qualifying variants (QV) and PRS-IPF in the PFFPR. 38
Supplementary Figure 5. Association between prevalence of qualifying variants (QV) and PRS-IPF (after excluding the MUC5B locus) in the PFFPR. 39

Supplementary Figure 6. Association between the prevalence of qualifying variants (QV) and PRS-TL in the PFFPR. 40
Supplementary Figure 7. Association between prevalence of qualifying variants (QV) in telomere and non-telomere genes and PRS-TL in the PFFPR. 41
Supplementary Figure 8. Association between prevalence of qualifying variants (QV) in telomere genes and PRS-TL in the PFFPR. 42
Supplementary Figure 9. Kaplan-Meier survival analysis for qualifying variants (QV) (per gene and group of genes) and the *MUC5B* risk allele in the PFFPR. p-values for the log-rank test are shown. 43
Supplementary Figure 10. Qualifying variants (QV) effect on survival in the PFFPR (excluding carriers of QV within PARN). 44
Supplementary Figure 11. **Alternative qualifying variants (QV) classifications and effects on survival in the PFFPR**. 45
Supplementary Figure 12. Association between PRS-TL tertiles and survival in the PFFPR. 46
Supplementary Figure 13. Association between high and low PRS-TL and survival in the PFFPR. 47
Supplementary Figure 14. Association of PRS-IPF (after excluding the MUC5B locus) and survival in the PFFPR. Kaplan-Meier analysis showing p-values for the log-rank test. 48
Supplementary Figure 15. Associations between PRS-IPF and *MUC5B* rs35705950 genotypes with survival among carriers and non-carriers of qualifying variants (QV) in the PFFPR. 49
Supplementary Figure 16. Association between prevalence of qualifying variants (QV) and PRS-IPF in PROFILE.. 50
Supplementary Figure 17. **Kaplan-Meier survival analysis for qualifying variants (QV) (per gene and group PF genes) and the MUC5B risk allele in PROFILE. p-values for the log-rank test are shown.** 51
Supplementary references 52

Supplementary methods

Description of study cohorts

The Pulmonary Fibrosis Foundation Patient Registry (PFFPR) is a large multicentre based registry that collects baseline and longitudinal demographic and clinical information about well-characterized patients with interstitial lung diseases (ILD) in the United States since March 2016 to allow retrospective and prospective research[1]. In addition, the PFFPR major objective is to apply blood-based omics technologies (whole-genome sequencing [WGS], proteomic analysis, and transcriptional profiling) on blood samples from patients to study molecular markers of the onset or progression of diseases. Patients aged ≥18 years old who has ILD diagnosed and had not undergone lung transplantation were recruited from approximately 42 USA sites selected primarily from the familial pulmonary fibrosis (FPF) Care Center Network. They were followed for the progression of the disease through the lifetime of the PFFPR or the patient until the patient receives lung transplant. More details of the PFFPR including inclusion and exclusion criteria as well as collected clinical variables are described elsewhere[1]. The PFFPR cohort includes 1317 individuals with ILD for whom WGS data are available. For this study, we included the 917 PFFPR patients with a definitive IPF diagnosis. Family history was available for all of them although no genetic causes were previously assessed. After the quality control procedures, 888 of those patients remained in the study.

The PROFILE is a UK large, prospective, multicentre, longitudinal study conducted on patients with fibrotic ILD[2,3]. The cohort includes 541 patients with IPF or idiopathic non-specific interstitial pneumonia aged 18-85 recruited from tertiary specialist ILD and from local secondary care hospitals. Blood samples for genomic analysis were collected and they were followed for disease progression through 3 years. After quality control steps, the second stage of the study included 472 patients with a confirmed diagnosis of IPF.

Baseline characteristics of the PFFPR and the PROFILE cohorts are listed in **Supplementary Table 1**.

Supplementary sequencing and bioinformatics analysis methods

In the PFFPR, library preparation and sequencing were performed by Psomagen (Rockville, MD). Genomic DNA libraries were prepared using the TruSeq DNA PCR Free kit (Illumina Inc.) and sequenced on an Illumina NovaSeq 6000 instrument (Illumina Inc.) with 150 bp paired-end reads at an average depth of 30X. At least 80% of the genome was covered by ≥20 reads, and ≥90% was covered by ≥10 reads. Whole genome sequencing (WGS) was processed using the Illumina DRAGEN Bio-IT Platform Germline Pipeline v3.10.4 (Illumina Inc.) using the Illumina DRAGEN Multigenome Graph hg38 as the reference genome. Only variants with a "PASS" filter were included in subsequent analyses.

To obtain estimates of telomere length (TL) from WGS BAM files (WGS-TL) we used TelSeq 0.0.2[4]. A hexamer repeat threshold of 12 was selected as described elsewhere[5].

For the PROFILE cohort, WGS was performed at Human Longevity Inc. using the Illumina NovaSeq 6000 system with 150 pb paired-end reads. Coverage of at least 10X was achieved in over 98% of the Consensus Coding Sequence Release 22 (CCDS), with an average read depth of 42X across the CCDS as described previously [6]. Sequences were processed using the Illumina DRAGEN Bio-IT Platform Germline Pipeline v3.0.7, with the GRCh38 as the reference genome.

In both cohorts, several quality control (QC) analyses were performed: (i) detection of QC outliers, (ii) the kinship between patients, (iii) sample cross-contamination, and (iv) sex discordance. We used a combination of DRAGEN metrics, and assessments with PLINK v1.90b6.24[2,3], SCE-VCF v0.1.2 (https://github.com/HTGenomeAnalysisUnit/SCE-VCF), Somalier v0.2.19[2,3], and KING v2.3.2[6].

Detection of QC outliers: Based on PLINK analysis, we detected abnormal heterozygosity rate and genotyping call rate to infer potential sample contaminations and/or a low DNA concentration. A heterozygosity rate value ± 3 standard deviations from the mean and/or genotype call rate <0.95 were considered as outliers.

Kinship between patients: We detected duplicates or monozygotic twins, and first-degree kinship relationships with three different tools: we considered two samples as duplicates or obtained from monozygotic twins if a PI_HAT value was >0.9 for PLINK, a Somalier relatedness value >0.9, and a KING kinship coefficient >0.354. We considered as first-degree relatives a PI_HAT in the range of 0.4-0.6 for PLINK, a Somalier relatedness value in the range of 0.4-0.6, and a KING kinship coefficient in the range of 0.177-0.354. We found a complete consensus among these tools in the cohort. Second-degree relatives were not detected.

Sample cross-contamination: We used the "*estimated_sample_contamination*" parameter from DRAGEN metrics to exclude samples with evidence ≥2% of contamination. We also used SCE-VCF tool, which estimates contamination from VCF files using the CHARR method[7], based on the recommended thresholds to consider a sample as contaminated (CHARR > 0.03 and INCONSISTENT_AB_HET_RATE > 0.15). We found a complete consensus among these tools in the identification of potential sample contamination in the PFF-PR. For PROFILE, we only relied on SCE-VCF for the sample cross-contamination inference.

Sex discordance: Biological sex inference from genetic data was obtained with Somalier following recommendations. For that we compared the scaled mean depth on X and Y chromosomes for 365 and 17 genomic positions, respectively. Sex discordance, identified by comparing the genetically inferred sex with that recorded, was also used to exclude patients from the study. In the PFF-PR, a female was identified as a possible X0 aneuploid due to the low number of heterozygous sites on the X chromosome and was excluded from the analysis.

Identification of qualifying variants (QVs) in monogenic adult-onset PF genes

The identification of QVs was restricted to 13 PF genes classified as telomere related and non-telomere related **(Supplementary Table 2)**. With the exception of *SPDL1*, and *KIF15*, this list includes genes with a known dominant inheritance pattern (presuming that QVs in these genes would have higher penetrance) and genes that are commonly found in familial IPF cohorts, although they also occur in sporadic cases [7].

KIF15 and *SPDL1* were incorporated to the list as recent large scale sequencing studies identified them as PF-related genes [6,8,9]. Both genes are critical for mitosis, pointing to a novel, non-telomeric mechanism underlying IPF. Rare deleterious variants in *KIF15* and three telomere genes (*TERT, PARN* and *RTEL1*) have been previously associated with IPF risk, early onset, and progression to early-age lung transplantation or death [9]. In *SPDL1,* a rare missense variant was confirmed as a new IPF risk allele, although carriers did not exhibit distinct clinical features [6].

Variants in these genes were filtered based on read depth (DP) <10, mapping quality (MQ) <50, or the percentage of missing genotypes (FMISS) >0.05 in the cohort. The remaining variants were annotated using the Variant Effect Predictor tool v109.3 [10]. Variants with a global allele frequency (AF) >0.0005 in gnomAD v2.1 were excluded from the study. For our analyses, we retained protein-truncating variants (including frameshift, stop-gained, start-loss, and splicing variants) and missense variants with a CADD >15.

For the non-coding RNA gene *TERC*, due to the difficulty in predicting functional effects in non-coding genes, variants were considered for the analysis if their global population AF was <0.0005 and they were annotated by ClinVar as pathogenic (P), likely pathogenic (LP), or of uncertain significance (VUS).

Principal components of genetic heterogeneity in the cohorts

Principal components (PC) were calculated after excluding single nucleotide polymorphisms (SNPs) with a minor allele frequency (MAF) <0.01 from WGS data, using BCFtools (https://samtools.github.io/bcftools/bcftools.html). Genotyping QC was then performed using PLINK v.1.9. First, SNPs with a genotyping call rate (CR) <95% or those deviating significantly from Hardy-Weinberg equilibrium (HWE) ($p<1.0x10^{-6}$) were removed. After linkage disequilibrium pruning (indep-pairwise 100 5 0.01), the main PCs of genetic variation were calculated based on 110,951 independent SNPs in the PFFPR and 143,214 independent SNPs in PROFILE **(Supplementary Figure 1)**.

Estimation of polygenic risk scores (PRS)

We derived PRS-IPF from 19 previously published genome-wide significant IPF variants **(Supplementary Table 6)** using PRSice-2 [11]. Briefly, PRS were calculated as the number of risk alleles carried by each individual, multiplied by the effect size of the variant as described in the GWAS study [11] summed across all variants included in the score:

$$\text{PRS} = \sum_{i=1}^{n} \beta\text{i}\text{Gi}$$

where βi is the OR (in the case of binary traits) from variant i, Gi represents the number of risk alleles carried at the variant i and n represents the conditionally independent signals identified elsewhere. Raw polygenic scores were then standardized as z-scores using the following formula:

$$PRSz = \frac{PRs - Mean\ (PRS)}{standard\ deviations\ (PRS)}$$

The same methodology was used to derive PRS for TL (PRS-TL) based on the 20 common variants that were previously found associated with leukocyte TL[12] **(Supplementary Table 7)**. In this case, since TL is a quantitative trait, βi is represented by beta coefficients in the PRS formula.

Supplementary results

Prevalence of QVs in the PFFPR

A total of 131 QVs were identified in monogenic adult-onset PF genes in 144 patients. Most patients (96.5%, 139 of 144) carried a single QV, while five patients (3.5% of the QV carriers) had two or more QVs, including combinations such as *NAF1/TERT, KIF15/RTEL1, TERT/SPDL1, TINF2/TERT/RTEL1*, and *RTEL1/RTEL1*. Consistent with previous studies, the prevalence of QVs was higher in patients with a familial history of disease (27.3%, 48 of 176) compared to those with sporadic disease (13.5%, 96 of 712) (p=3.08x10^{-5}). Most QVs were in telomere genes (75.6%, 99 of 131), while nearly a quarter were found in non-telomere genes (24.4%, 32 of 131). The highest number of QVs were identified in telomere-related genes including *RTEL1* (25.2%, 33 of 131), *TERT* (23.7%, 31 of 131), and *PARN* (12.2%, 16 of 131). These genes also had the highest proportion of P/LP variants (31.0%, 18 of 58, 31.0%, 18 of 58, and 20.7%,12 of 58, respectively). In total, 42.7% (56 of 131) of QVs were previously annotated in ClinVar as VUS, LP, or P.

Prevalence of QVs in PROFILE

Using the same classification of QVs as in PFFPR, the diagnostic yield of finding a QVs in PROFILE was 15.67 % (74 of 472) (95% CI=12.4-19.0%) **(Supplementary Table 4)**. The genes with the highest burden of QVs were: *RTEL1* (20.5%, 15 of 73), *TERT* (15.1%, 11 of 73), and *PARN* (17.8%, 13 of 73) **(Supplementary Figure 2B-D)**. The prevalence of QVs among carriers of the risk *MUC5B* genotype (rs35705950-T) was lower (14.97%, 44 of 294) than among those carrying the protective GG genotype (16.85%, 30 of 178), although the difference was not statistically significant (p=0.60). We observed the same effect direction as in PFFPR when assessing the association between the lower PRS-IPF tertile and reduced survival (HR=1.49, 95% CI=0.14-1.95, p=3.1x10^{-3}).

Supplementary Tables

Supplementary Table 1. Baseline characteristics and outcomes of IPF patients from stage one and stage two cohorts.

Characteristics	PFFPR (n=888)*	PROFILE (n=472)$
Age, yr, mean (SD)	71.02 (7.8)	70.65 (7.9)
Male, n (%)	676 (76.1)	366 (77.5)
Self-reported ethnicity, n (%)		
Unknown	17	
Asian	23	-
Black	10	
White	838	
Ever smoker, n, (%)	571 (64.3)	326 (69.1)
Familial history, n, (%)	176 (19.8)	-
FVC% predicted, mean (SD)	67.74 (16.8)	78.97 (19.01)
DLCO% predicted, mean (SD)	29.3 (4.8)	44.97 (14.98)
Carriers of QVs	144	74
Dead at 60 months, n, (%)	37 (25.7)	53 (71.6)
Transplant at 60 months, n (%)	22 (15.3)	-
Non-carriers of QVs	744	398
Dead, n, at 60 months (%)	163 (21.9)	239 (60.1)
Transplant at 60 months, n (%)	70 (9.4)	-
Median survival in years (IQR)	4.86 (3.31-6.93)	3.30 (1.74-5.71)
MUC5B genotype with risk allele	622 (70)	294 (62.3)

PFFPR, The Pulmonary Fibrosis Foundation Patient Registry; SD, standard deviation; FVC, forced vital capacity; DLCO, diffusing capacity of the lungs for carbon monoxide; IQR=interquartile range; QVs, qualifying variant. *Missing data: FVC predicted (n=41) and DLCO predicted (n=68); $Missing data: FVC predicted (n=12) and DLCO predicted (n=50).

Supplementary Table 2. Regions of interest (ROIs) for qualifying variants annotations in hg38.

Chromosome	Gene	Start	End
5	*TERT*	1,253,047	1,295,168
3	*TERC*	169,764,420	169,765,160
14	*TINF2*	24,238,186	24,242,763
X	*DKC1*	154,762,642	154,777,789
20	*RTEL1*	63,657,710	63,696,353
16	*PARN*	14,435,600	14,632,828
4	*NAF1*	163,109,973	163,166,990
12	*ZCCHC8*	122,471,500	122,501,032
8	*SFTPC*	22,156,813	22,164,579
10	*SFTPA2*	79,555,752	79,560,507
10	*SFTPA1*	79,610,839	79,615,555
5	*SPDL1*	169,583,536	169,604,878
3	*KIF15*	44,761,621	44,873,476

.

Supplementary Table 3. Qualifying variants identified in the PFFPR.

Variant	Gene	HGVSc	HGVSp	Effect	GnomADg AF	CADD	Alpha Missense	Condel	PrimateAI	REVEL	ClinVar	ACMG Class
10_79557337_T_A	*SFTPA2*	c.619A>T	p.Asn207Tyr	missense	.	23	0.3054	deleterious (0.998)	0.47	0.16	.	VUS
10_79614091_G_A	*SFTPA1*	c.725G>A	p.Arg242Gln	missense	.	23.4	0.3594	deleterious (1.000)	0.54	0.348	uncertain significance	VUS
12_122473969_C_T	*ZCCHC8*	c.938G>A	p.Gly313Asp	missense	0.000368	23.5	.	deleterious (1.000)	0.64	0.115	uncertain significance	VUS
12_122481951_A_G	*ZCCHC8*	c.155T>C	p.Val52Ala	missense	.	24.6	.	deleterious (0.893)	0.54	0.117	.	VUS
12_122483508_G_A	*ZCCHC8*	c.557C>T	p.Pro186Leu	missense	.	28	0.8556	deleterious (0.999)	0.73	.	pathogenic	P
12_122498861_G_A	*ZCCHC8*	c.208C>T	p.Leu70Phe	missense	.	25.8	0.3853	deleterious (0.887)	0.70	.	.	VUS
12_122498866_T_C	*ZCCHC8*	c.203A>G	p.Gln68Arg	missense	.	20.4	0.0665	Neutral (0.405)	0.51	.	.	VUS
14_24240544_G_T	*TINF2*	c.936C>A	p.Tyr312Ter	stop gained	5.92E-05	33	.	.	.	.	uncertain significance&not provided	LP
14_24240636_G_A	*TINF2*	c.844C>T	p.Arg282Cys	missense	.	30	0.1866	deleterious (0.976)	0.51	0.878	pathogenic	P
14_24241308_G_A	*TINF2*	c.403C>T	p.Leu135Phe	missense	0.0001192	23.8	0.2901	deleterious (0.924)	0.46	0.213	uncertain significance	LP
16_14447001_CCT_C	*PARN*	c.1566 1567del	p.Glu524AspfsTer5	frameshift	5.26E-05	23.1	.	.	.	.	likely pathogenic&pathogenic	P
16_14482829_T_C	*PARN*	c.1298-2A>G	.	splice acceptor	.	35	.	.	.	.	.	LP
16_14531059_T_C	*PARN*	c.1377A>G	p.Ter459TrpextTer19	stop lost& NMD transcript	.	5.885	.	.	.	.	.	VUS
16_14554139_C_T	*PARN*	c.1148G>A	p.Arg383His	missense	1.97E-05	31	.	deleterious (1.000)	0.82	0.564	uncertain significance	LP
16_14554150_CACT_C	*PARN*	c.1136-2 1136del	.	splice acceptor&coding sequence	.	.	.	.	.	.	.	LP
16_14555675_G_C	*PARN*	c.1114C>G	p.Leu372Val	missense	9.87E-05	20.8	.	deleterious (0.567)	0.70	0.086	uncertain significance	VUS
16_14555710_C_T	*PARN*	c.1080-1G>A	.	splice acceptor	.	34	.	.	.	.	.	LP
16_14582222_T_C	*PARN*	c.968A>G	p.Tyr323Cys	missense	0.0001051	28.9	.	deleterious (1.000)	0.87	0.541	uncertain significance	LP
16_14582237_G_A	*PARN*	c.953C>T	p.Ala318Val	missense	.	32	.	deleterious (1.000)	0.84	0.789	.	LP

Supplementary Table 3. Qualifying variants identified in the PFFPR.

Variant	Gene	HGVSc	HGVSp	Effect	GnomADg AF	CADD	Alpha Missense	Condel	PrimateAI	REVEL	ClinVar	ACMG Class
16_14582250_G_A	*PARN*	c.940C>T	p.Gln314Ter	stop gained	.	41	.	.	.	.	.	P
16_14584424_T_C	*PARN*	c.823-2A>G	.	splice acceptor	.	34	.	.	.	.	likely pathogenic&path ogenic	P
16_14584781_T_C	*PARN*	c.790A>G	p.Thr264Ala	missense	.	27	.	deleterious (0.948)	0.76	0.666	.	VUS
16_14599924_T_TG ACTCTAGAAATG ATTTCTA	*PARN*	c.636 637insTAGAAATC ATTTCTAGAGTC	p.Ile213Ter	stop gained& frameshift	.	.	.	.	.	.	.	P
16_14610732_C_A	*PARN*	c.283G>T	p.Ala95Ser	missense	0.0003614	22.3	.	deleterious (0.479)	0.45	0.217	uncertain significance	VUS
16_14617596_G_A	*PARN*	c.199C>T	p.Arg67Ter	stop gained	1.32E-05	39	.	.	.	.	pathogenic	P
16_14629597_C_A	*PARN*	c.97G>T	p.Gly33Ter	stop gained& splice region	.	48	.	.	.	.	.	LP
20_63659405_G_A	*RTEL1*	c.3G>A	p.Met1?	start lost	.	28.3	.	deleterious (0.984)	.	.	.	LP
20_63662544_A_G	*RTEL1*	c.-274-2A>G	.	splice acceptor	.	32	.	.	0.56	.	.	P
20_63662590_A_G	*RTEL1*	c.590A>G	p.His197Arg	missense	6.57E-06	25.5	.	deleterious (0.541)	0.49	0.702	.	VUS
20_63667494_A_T	*RTEL1*	c.790A>T	p.Asn264Tyr	missense	4.60E-05	27.3	.	deleterious (0.500)	0.55	0.297	uncertain significance	LP
20_63672560_G_A	*RTEL1*	c.35G>A	p.Arg12His	missense	.	26.1	.	deleterious (1.000)	0.64	0.669	uncertain significance	LP
20_63674034_C_T	*RTEL1*	c.191C>T	p.Thr64Ile	missense	0.0001448	20.9	.	deleterious (0.480)	0.41	0.115	uncertain significance	LP
20_63678280_G_T	*RTEL1*	c.302G>T	p.Arg101Leu	missense	.	17.73	.	deleterious (0.487)	0.32	0.407	uncertain significance	VUS
20_63678292_C_A	*RTEL1*	c.314C>A	p.Ala105Asp	missense	.	24.4	.	deleterious (0.774)	0.50	0.68	.	VUS
20_63685559_GGT TCCCCAGCAGGG CTGGGGGCCTTA CAGTCCTATAAG GTAGGGGCCACC TCCAGGAGGCAG GTGGAGGGCAGC CCTT_G	*RTEL1*	c.567 597+47del	.	splice donor& splice donor 5th base& coding sequence&int ron	.	.	.	.	.	.	.	P

Supplementary Table 3. Qualifying variants identified in the PFFPR.

Variant	Gene	HGVSc	HGVSp	Effect	GnomADg AF	CADD	Alpha Missense	Condel	PrimateAI	REVEL	ClinVar	ACMG Class
20_63685825_C_T	*RTEL1*	c.632C>T	p.Thr211Met	missense	0.0004336	16.46	.	deleterious (0.629)	0.33	0.251	uncertain significance	VUS
20_63687740_C_T	*RTEL1*	c.782C>T	p.Pro261Leu	missense	6.57E-06	27	.	deleterious (1.000)	0.69	0.759	uncertain significance&pathogenic	LP
20_63687765_G_T	*RTEL1*	c.807G>T	p.Met269Ile	missense	6.57E-06	24.6	.	deleterious (0.534)	0.62	0.74	pathogenic&likely pathogenic	P
20_63688016_G_A	*RTEL1*	c.892G>A	p.Asp298Asn	missense	.	25.1	.	deleterious (0.984)	0.54	0.378	.	VUS
20_63688384_C_T	*RTEL1*	c.1051C>T	p.Arg351Trp	missense&splice region	6.58E-06	34	.	deleterious (0.976)	0.47	0.691	uncertain significance	LP
20_63688572_T_A	*RTEL1*	c.1098T>A	p.Phe366Leu	missense	.	23	.	deleterious (0.914)	0.69	0.711	.	LP
20_63689539_G_A	*RTEL1*	c.1247G>A	p.Arg416His	missense	6.57E-06	29.9	.	deleterious (1.000)	0.58	0.88	.	LP
20_63689566_A_AC	*RTEL1*	c.1280dup	p.Arg428ThrfsTer15	frameshift	.	.	.	.	.	.	.	P
20_63689807_A_T	*RTEL1*	c.1414A>T	p.Ile472Phe	missense	.	23.7	.	deleterious (0.999)	0.59	0.856	.	LP
20_63690100_G_A	*RTEL1*	c.1486G>A	p.Asp496Asn	missense	1.31E-05	22.7	.	deleterious (0.505)	0.56	0.296	uncertain significance	LP
20_63690110_C_T	*RTEL1*	c.1496C>T	p.Ala499Val	missense	.	24.6	.	deleterious (0.725)	0.48	0.626	.	VUS
20_63690206_G_A	*RTEL1*	c.1592G>A	p.Arg531Gln	missense	5.26E-05	19.86	.	deleterious (0.513)	0.37	0.055	uncertain significance	LP
20_63691777_G_C	*RTEL1*	c.1923G>C	p.Glu641Asp	missense	.	15.27	.	Neutral (0.432)	0.40	0.078	.	VUS
20_63692883_A_C	*RTEL1*	c.2062A>C	p.Asn688His	missense	6.57E-06	17.53	.	deleterious (0.692)	0.39	0.072	uncertain significance	VUS
20_63693145_TTC_T	*RTEL1*	c.2187 2188del	p.Phe729LeufsTer9	frameshift	.	.	.	.	.	.	.	LP
20_63693148_T_C	*RTEL1*	c.2188T>C	p.Tyr730His	missense	.	23.1	.	deleterious (0.889)	0.53	0.188	.	VUS
20_63693161_G_A	*RTEL1*	c.2201G>A	p.Arg734Gln	missense	1.32E-05	27.3	.	deleterious (0.924)	0.45	0.293	uncertain significance	VUS
20_63693208_G_A	*RTEL1*	c.2248G>A	p.Gly750Arg	missense	1.32E-05	25.1	.	deleterious (0.999)	0.47	0.332	.	VUS
20_63693211_C_T	*RTEL1*	c.2251C>T	p.Arg751Ter	stop gained	7.24E-05	39	.	.	.	.	pathogenic	P
20_63693220_G_C	*RTEL1*	c.2260G>C	p.Gly754Arg	missense	.	19.87	.	deleterious (0.940)	0.29	0.104	.	VUS
20_63693223_T_A	*RTEL1*	c.2263T>A	p.Tyr755Asn	missense	.	17.05	.	deleterious (0.497)	0.29	0.094	.	VUS

Supplementary Table 3. Qualifying variants identified in the PFFPR.

Variant	Gene	HGVSc	HGVSp	Effect	GnomADg AF	CADD	Alpha Missense	Condel	PrimateAI	REVEL	ClinVar	ACMG Class
20_63693226_C_T	*RTEL1*	c.2266C>T	p.Arg756Trp	missense	9.21E-05	19.07	.	deleterious (0.675)	0.21	0.086	uncertain significance	VUS
20_63693247_C_T	*RTEL1*	c.2287C>T	p.Arg763Ter	stop gained	8.55E-05	36	.	.	.	.	pathogenic/ likely pathogenic&pathogenic	P
20_63694956_G_A	*RTEL1*	c.2656G>A	p.Asp886Asn	missense	6.57E-06	18.23	.	deleterious (0.486)	0.35	0.305	uncertain significance	VUS
3_169764646_G_A	*TERC*	n.402C>T	.	non coding transcript exon	1.31E-05	19.94	.	.	.	.	uncertain significance	VUS
3_169764856_G_A	*TERC*	n.192C>T	.	non coding transcript exon	1.97E-05	21.1	.	.	.	.	uncertain significance	VUS
3_169764862_G_A	*TERC*	n.186C>T	.	non coding transcript exon	3.28E-05	16.88	.	.	.	.	uncertain significance	VUS
3_169764930_G_A	*TERC*	n.118C>T	.	non coding transcript exon	.	20.4	.	.	.	.	uncertain significance	VUS
3_44775328_A_G	*KIF15*	c.137A>G	p.Glu46Gly	missense	.	25.4	0.0909	deleterious (0.666)	0.41	0.193	.	VUS
3_44786440_A_G	*KIF15*	c.505A>G	p.Ile169Val	missense	6.57E-06	22.8	0.0816	deleterious (0.572)	0.66	0.431	.	VUS
3_44786480_C_T	*KIF15*	c.545C>T	p.Ser182Leu	missense	0.0001907	25.7	0.1035	deleterious (0.995)	0.70	0.525	.	VUS
3_44800332_A_T	*KIF15*	c.1117A>T	p.Thr373Ser	missense	5.91E-05	22.9	0.1669	deleterious (0.500)	0.77	0.103	.	VUS
3_44801488_G_A	*KIF15*	c.1261G>A	p.Ala421Thr	missense	6.58E-06	26.1	0.4025	deleterious (0.924)	0.50	0.23	.	VUS
3_44802841_T_C	*KIF15*	c.1537T>C	p.Tyr513His	missense	.	27	0.8168	deleterious (0.998)	0.83	0.342	.	LP
3_44810864_A_C	*KIF15*	c.1990A>C	p.Lys664Gln	missense	.	25.3	0.1939	deleterious (0.922)	0.55	0.183	.	VUS
3_44815078_T_C	*KIF15*	c.2549+2T>C	.	splice donor	.	33	.	.	.	.	.	LP
3_44828239_A_G	*KIF15*	c.2882A>G	p.Glu961Gly	missense	0.0003678	25.3	0.0851	deleterious (0.642)	0.33	0.075	.	VUS
3_44838278_G_C	*KIF15*	c.3175G>C	p.Asp1059His	missense	0.0003746	26.7	0.4345	deleterious (0.745)	0.60	0.1	.	VUS
3_44840427_G_T	*KIF15*	c.3391G>T	p.Val1131Leu	missense	.	21.9	0.2215	neutral (0.449)	0.63	0.049	.	VUS

Supplementary Table 3. Qualifying variants identified in the PFFPR.

Variant	Gene	HGVSc	HGVSp	Effect	GnomADg AF	CADD	Alpha Missense	Condel	PrimateAI	REVEL	ClinVar	ACMG Class
3_44840456_G_C	*KIF15*	c.3420G>C	p.Gln1140His	missense&splice region	1.32E-05	36	0.2366	deleterious (0.968)	0.42	0.138	.	VUS
3_44841128_C_G	*KIF15*	c.3475C>G	p.Gln1159Glu	missense	0.000138	22.1	0.0983	deleterious (0.551)	0.50	0.149	.	VUS
3_44851920_G_T	*KIF15*	c.3940G>T	p.Glu1314Ter	stop gained	0.000138	43	.	.	.	.	.	LP
3_44852694_G_A	*KIF15*	c.4126G>A	p.Glu1376Lys	missense	5.93E-05	29.9	0.1411	deleterious (0.881)	0.61	0.174	.	VUS
4_163127016_G_GT	*NAF1*	c.1132dup	p.Thr378AsnfsTer7	frameshift	0.0002234	0.94	.	.	.	.	.	LP
4_163129120_T_C	*NAF1*	c.1262A>G	p.Gln421Arg	missense	1.32E-05	22.2	0.0842	deleterious (0.517)	0.37	0.025	.	VUS
4_163129217_T_C	*NAF1*	c.1165A>G	p.Arg389Gly	missense	.	17.2	0.0841	deleterious (0.554)	0.40	0.16	.	VUS
4_163133221_T_G	*NAF1*	c.966A>C	p.Glu322Asp	missense	0.0004073	24.2	0.3312	deleterious (0.940)	0.48	0.144	.	VUS
4_163140334_A_C	*NAF1*	c.767T>G	p.Phe256Cys	missense	.	25.8	0.9299	deleterious (1.000)	0.72	0.674	.	VUS
4_163164384_C_T	*NAF1*	c.373G>A	p.Asp125Asn	missense	0.0002104	24.9	0.1694	deleterious (0.530)	0.84	0.145	.	VUS
5_1253777_G_T	*TERT*	c.3350C>A	p.Ala1117Asp	missense	.	18.97	0.1141	deleterious (0.711)	0.45	0.349	uncertain significance	LP
5_1253804_G_A	*TERT*	c.3323C>T	p.Pro1108Leu	missense	2.63E-05	20.7	0.1418	deleterious (0.544)	0.48	0.384	uncertain significance&likely benign	LP
5_1254406_C_T	*TERT*	c.3257G>A	p.Arg1086His	missense	0.0001971	21	0.0838	deleterious (0.816)	0.26	0.248	uncertain significance	LP
5_1254407_G_A	*TERT*	c.3256C>T	p.Arg1086Cys	missense	.	16.75	0.1256	deleterious (0.837)	0.29	0.365	.	VUS
5_1255344_G_A	*TERT*	c.3100C>T	p.Arg1034Cys	missense	.	20.4	0.1462	deleterious (0.934)	0.23	0.349	uncertain significance	LP
5_1258638_AC_A	*TERT*	c.2991del	p.Cys998AlafsTer50	frameshift	.	.	.	.	.	.	pathogenic	P
5_1260497_G_A	*TERT*	c.2947C>T	p.His983Tyr	missense	.	25.5	0.2451	deleterious (0.957)	0.45	0.439	uncertain significance&likely pathogenic	LP
5_1260574_C_G	*TERT*	c.2870G>C	p.Ser957Thr	missense	.	25.1	0.2679	deleterious (0.994)	0.54	0.287	.	VUS
5_1264461_G_A	*TERT*	c.2786C>T	p.Pro929Leu	missense	.	22.6	0.2786	deleterious (0.821)	0.78	0.838	.	VUS
5_1264485_T_G	*TERT*	c.2762A>C	p.Gln921Pro	missense	.	24.4	0.1065	deleterious (0.944)	0.56	0.641	.	VUS
5_1268527_G_A	*TERT*	c.2575C>T	p.Arg859Trp	missense	.	25.4	0.1242	deleterious (0.930)	0.21	0.344	uncertain significance	LP

Supplementary Table 3. Qualifying variants identified in the PFFPR.

Variant	Gene	HGVSc	HGVSp	Effect	GnomADg AF	CADD	Alpha Missense	Condel	PrimateAI	REVEL	ClinVar	ACMG Class
5_1268573_G_C	*TERT*	c.2529C>G	p.Ser843Arg	missense	.	24	0.9856	deleterious (0.997)	0.48	0.758	.	LP
5_1268586_G_A	*TERT*	c.2516C>T	p.Thr839Met	missense	.	26.1	0.305	deleterious (1.000)	0.42	0.716	uncertain significance	VUS
5_1278686_GA_G	*TERT*	c.2240del	p.Val747AlafsTer20	frameshift	.	.	.	.	.	.	pathogenic	P
5_1278692_GGCAT ACCGAC_G	*TERT*	c.2225 2234del	p.Arg742ProfsTer22	frameshift	.	.	.	.	.	.	.	LP
5_1278762_T_C	*TERT*	c.2131-2A>G	.	splice acceptor&NMD transcript	.	24.7	.	.	0.32	.	uncertain significance	VUS
5_1279311_G_A	*TERT*	c.2110C>T	p.Pro704Ser	missense	4.60E-05	16.1	0.1022	deleterious (0.962)	0.32	0.619	uncertain significance&pathogenic	P
5_1279328_C_T	*TERT*	c.2093G>A	p.Arg698Gln	missense	.	23.7	0.0997	deleterious (0.843)	0.46	0.406	.	VUS
5_1279340_A_T	*TERT*	c.2081T>A	p.Val694Glu	missense	6.57E-06	17.01	0.7432	deleterious (0.998)	0.58	0.681	uncertain significance	LP
5_1279352_C_G	*TERT*	c.2069G>C	p.Trp690Ser	missense	.	25.1	0.96	deleterious (0.995)	0.66	0.89	.	LP
5_1279407_G_A	*TERT*	c.2014C>T	p.Arg672Cys	missense	0.0002169	22.8	0.1047	deleterious (0.897)	0.40	0.44	uncertain significance	VUS
5_1279416_G_A	*TERT*	c.2005C>T	p.Arg669Trp	missense	1.31E-05	22.8	0.2692	deleterious (0.649)	0.66	0.718	uncertain significance	VUS
5_1279464_G_A	*TERT*	c.1957C>T	p.Arg653Cys	missense	2.63E-05	17.4	0.1146	Neutral (0.456)	0.27	0.279	uncertain significance	VUS
5_1280313_G_A	*TERT*	c.1795C>T	p.Arg599Trp	missense	6.57E-06	24.9	0.1544	deleterious (0.993)	0.38	0.454	.	VUS
5_1282594_C_T	*TERT*	c.1604G>A	p.Arg535His	missense	6.57E-06	22.2	0.1095	deleterious (0.762)	0.37	0.418	uncertain significance	LP
5_1282609_G_A	*TERT*	c.1589C>T	p.Pro530Leu	missense	1.32E-05	25	0.279	deleterious (0.981)	0.53	0.583	uncertain significance	VUS
5_1293588_C_T	*TERT*	c.1298G>A	p.Gly433Asp	missense	7.88E-05	16.51	0.0718	deleterious (0.882)	0.56	0.289	likely benign	VUS
5_1294470_A_C	*TERT*	c.416T>G	p.Leu139Arg	missense	.	24	0.1687	deleterious (0.962)	0.87	0.639	uncertain significance	LP
5_1294549_C_CG	*TERT*	c.336dup	p.Glu113ArgfsTer79	frameshift	6.58E-06	16.1	.	.	.	.	pathogenic	P
5_1294619_G_C	*TERT*	c.267C>G	p.Cys89Trp	missense	.	22	0.7094	deleterious (0.996)	0.96	0.646	.	LP
5_1294913_G_A	*TERT*	c.77C>T	p.Thr26Met	missense	6.58E-06	23.3	0.3319	deleterious (0.807)	0.93	0.301	uncertain significance	LP
5_169591225_G_A	*SPDL1*	c.336+1G>A	.	splice donor	.	33	.	.	.	.	.	LP

Supplementary Table 3. Qualifying variants identified in the PFFPR.

Variant	Gene	HGVSc	HGVSp	Effect	GnomADg AF	CADD	Alpha Missense	Condel	PrimateAI	REVEL	ClinVar	ACMG Class
5_169594287_C_T	*SPDL1*	c.674C>T	p.Ala225Val	missense	0.0001709	23	0.2653	deleterious (0.999)	0.54	0.233	.	VUS
5_169594592_A_G	*SPDL1*	c.802A>G	p.Met268Val	missense	3.94E-05	22.8	0.123	deleterious (0.984)	0.49	0.213	.	VUS
5_169594598_C_T	*SPDL1*	c.808C>T	p.Arg270Cys	missense	.	23.5	0.2067	deleterious (0.511)	0.51	0.179	.	VUS
5_169596559_A_G	*SPDL1*	c.892-2A>G	.	splice acceptor	0.0004009	35	.	.	.	.	likely benign	VUS
5_169599138_A_G	*SPDL1*	c.1303A>G	p.Lys435Glu	missense	0.0002167	25.4	0.3092	deleterious (0.811)	0.59	0.153	.	VUS
5_169599145_A_C	*SPDL1*	c.1310A>C	p.Lys437Thr	missense	.	27.3	0.6537	deleterious (0.930)	0.72	0.312	.	VUS
5_169601597_A_G	*SPDL1*	c.1642A>G	p.Arg548Gly	missense	.	22.4	0.0901	deleterious (0.567)	0.30	0.129	.	VUS
5_169604205_TA_T	*SPDL1*	c.*1del	.	frameshift&st op lost	1.31E-05	27.3	.	.	.	.	.	LP
8_22159082_G_T	*SFTPC*	n.358+1G>T	.	splice donor&non coding transcript	3.94E-05	18.25	.	.	.	.	.	VUS
8_22161838_G_A	*SFTPC*	c.10G>A	p.Gly4Ser	missense	3.29E-05	15.48	0.0978	Neutral (0.107)	0.55	0.198	uncertain significance	VUS
8_22162605_G_A	*SFTPC*	c.74G>A	p.Gly25Asp	missense	.	21.3	0.2176	deleterious (0.524)	0.41	0.282	.	VUS
8_22162681_T_G	*SFTPC*	c.150T>G	p.Ile50Met	missense	5.26E-05	22.4	0.2106	deleterious (0.967)	0.64	0.658	.	VUS
8_22163988_C_G	*SFTPC*	c.541C>G	p.Leu181Val	missense	0.0004007	23.9	0.1813	Deleterious (0.998)	0.53	0.65	conflicting interpretations of pathogenicity&li kely benign	VUS
8_22164440_C_A	*SFTPC*	c.451C>A	p.Pro151Thr	missense	0.0001314	17.12	.	.	.	0.047	uncertain significance	VUS
X_154764944_C_T	*DKC1*	c.62C>T	p.Ser21Leu	missense	4.45E-05	19.32	0.0607	Deleterious (0.490)	0.41	0.242	.	VUS

HGVS, Human Genome Variation Society; VUS, variant of uncertain significance; P, pathogenic; LP, likely pathogenic

Supplementary Table 4. Qualifying variants identified in PROFILE.

Variant	Gene	HGVSp	HGVSp	Effect	GnomADg AF	CADD	Alpha Missense	Condel	Primate AI	REVEL	ClinVar Sig	ACMG Class
5_1260499_C_T	*TERT*	c.2945G>A	p.Cys982Tyr	missense	4.01E-06	27.1	0.7986	Deleterious (1.000)	0.71	0.573	uncertain significance	VUS
5_1268521_C_T	*TERT*	c.2581G>A	p.Gly861Arg	missense &splice region	.	24.9	0.8094	Deleterious (0.861)	0.44	0.801	uncertain significance	LP
5_1268577_C_T	*TERT*	c.2525G>A	p.Cys842Tyr	missense	.	25	0.9447	Deleterious (0.998)	0.68	0.931	uncertain significance	LP
5_1271131_C_T	*TERT*	c.2456G>A	p.Arg819His	missense	8.06E-06	21.3	0.0834	deleterious (0.955)	0.29	0.312	uncertain significance	VUS
5_1271156_G_A	*TERT*	c.2431C>T	p.Arg811Cys	missense	0	23	0.1142	deleterious (0.954)	0.24	0.587	uncertain significance&pathogenic&likely pathogenic	LP
5_1272184_C_G	*TERT*	c.2382+1G>C		splice donor	.	32	.	.	.	.	likely pathogenic	LP
5_1279407_G_A	*TERT*	c.2014C>T	p.Arg672Cys	missense	5.19E-05	22.8	0.1047	Deleterious (0.897)	0.40	0.44	uncertain significance	VUS
5_1280244_G_A	*TERT*	c.1864C>T	p.Arg622Cys	missense	.	27.3	0.6064	Deleterious (0.988)	0.58	0.9	uncertain significance	LP
5_1282573_G_A	*TERT*	c.1625C>T	p.Ala542Val	missense	.	23.5	0.2754	Deleterious (0.962)	0.51	0.474	uncertain significance	VUS
5_1293522_T_A	*TERT*	c.1364A>T	p.His455Leu	missense	0	26.5	0.4173	Deleterious (0.999)	0.79	0.845	uncertain significance	LP
5_1294378_C_T	*TERT*	c.508G>A	p.Val170Met	missense	5.35E-06	25.3	0.6491	Deleterious (0.913)	0.89	0.686	uncertain significance&pathogenic	LP
16_14447001_CCT_C	*PARN*	c.1566_1567del	p.Glu524AspfsTer5	frameshift	.	23.1	.	.	.	.	likely pathogenic&pathogenic	P
16_14482683_TG_T	*PARN*	c.1441del	p.Gln481SerfsTer23	frameshift	4.02E-06	.	.	.	.	.	pathogenic	P
16_14482828_C_T	*PARN*	c.1298-1G>A		splice acceptor	4.50E-06	35	.	.	.	.	.	LP
16_14554109_T_C	*PARN*	c.1178A>G	p.Lys393Arg	missense	8.05E-06	24.1	.	deleterious (0.816)	0.76	0.172	.	VUS
16_14554139_C_T	*PARN*	c.1148G>A	p.Arg383His	missense	4.04E-06	31	.	deleterious (1.000)	0.82	0.564	uncertain significance	LP
16_14555652_A_G	*PARN*	c.1135+2T>C		splice donor	.	34	.	.	.	.	.	P
16_14593338_A_G	*PARN*	c.698T>C	p.Met233Thr	missense	.	25.8	.	deleterious (0.974)	0.78	0.277	.	VUS
16_14617640_A_G	*PARN*	c.155T>C	p.Ile52Thr	missense	2.01E-05	27.2	.	deleterious (1.000)	0.83	0.594	.	LP

Supplementary Table 4. Qualifying variants identified in PROFILE.

Variant	Gene	HGVSp	HGVSp	Effect	GnomADg AF	CADD	Alpha Missense	Condel	Primate AI	REVEL	ClinVar Sig	ACMG Class
16_14617651_C_T	*PARN*	c.145-1G>A		splice acceptor	.	33	.	.	.	.	.	LP
16_14627163_G_C	*PARN*	c.87C>G	p.Phe29Leu	missense	.	26.3	.	deleterious (0.999)	0.81	0.64	.	LP
16_14628170_GC_G	*PARN*	c.-7+1del		splice donor	.	.	.	.	.	.	.	P
16_14628181_C_G	*PARN*	c.168G>C	p.Lys56Asn	missense	2.43E-05	23.1	0.969	deleterious (0.895)	0.84	0.188	uncertain significance	VUS
16_14629675_C_T	*PARN*	c.-164-1G>A		splice acceptor	.	34	.	.	.	.	.	LP
8_22163546_GGT_G	*SFTPC*	c.435+12_435+13 del		splice donor	0.000588	24.5	.	.	.	.	.	LP
8_22163988_C_G	*SFTPC*	c.541C>G	p.Leu181Val	missense	0.000507	23.9	0.1813	deleterious (0.998)	0.53	0.65	conflicting interpretations of pathogenicity&likely benign	VUS
8_22164440_C_A	*SFTPC*	c.451C>A	p.Pro151Thr	missense	7.59E-05	17.12	.	.	.	0.047	uncertain significance	VUS
3_44784844_G_A	*KIF15*	c.362-1G>A		splice acceptor	5.72E-05	35	.	.	.	.	.	LP
3_44797853_C_T	*KIF15*	c.995C>T	p.Ala332Val	missense	7.97E-06	27	0.8287	deleterious (0.994)	0.81	0.796	.	LP
3_44801792_G_T	*KIF15*	c.1327G>T	p.Glu443Ter	stop gained	2.02E-05	38	.	.	.	.	.	LP
3_44801870_C_T	*KIF15*	c.1405C>T	p.Arg469Cys	missense	1.20E-05	29.6	0.13	deleterious (0.978)	0.60	0.576	.	VUS
3_44830971_G_A	*KIF15*	c.3124G>A	p.Asp1042Asn	missense	1.59E-05	24.5	0.0846	deleterious (0.736)	0.45	0.159	.	VUS
3_44841171_TG_T	*KIF15*	c.3520del	p.Glu1174AsnfsTer4	frameshift	3.98E-06	.	.	.	.	.	.	LP
3_44851858_G_A	*KIF15*	c.3878G>A	p.Arg1293Gln	missense	1.59E-05	26.1	0.1084	deleterious (1.000)	0.42	0.257	.	VUS
3_44851941_A_G	*KIF15*	c.3961A>G	p.Arg1321Gly	missense	7.99E-06	25.6	0.1275	deleterious (0.520)	0.36	0.103	.	VUS
20_63661942_C_T	*RTEL1*	c.394C>T	p.Arg132Cys	missense &splice region	3.98E-06	32	.	deleterious (0.574)	0.43	0.645	uncertain significance	VUS
20_63665301_G_A	*RTEL1*	n.235+1G>A		splice donor &non coding transcript	.	1.042	.	.	.	.	.	VUS
20_63679869_C_T	*RTEL1*	c.389C>T	p.Ala130Val	missense	2.01E-05	26.7	.	deleterious (0.917)	0.49	0.591	uncertain significance	LP

Supplementary Table 4. Qualifying variants identified in PROFILE.

Variant	Gene	HGVSp	HGVSp	Effect	GnomADg AF	CADD	Alpha Missense	Condel	Primate AI	REVEL	ClinVar Sig	ACMG Class
20_63679873_AG_A	*RTEL1*	c.394del	p.Ala132ProfsTer99	frameshift	.	.	.	.	.	.	.	LP
20_63680717_C_G	*RTEL1*	c.520C>G	p.Gln174Glu	missense &splice region	0.000595	24.4	.	deleterious (0.528)	0.50	0.426	uncertain significance	LP
20_63685567_AGCAGGGCTGGGGGCCTTACAGTCCTATAAGGTAGGGGCCACCTCCAGGAGGCAGGTGGAGGGCAGCCCTTGTTCCCCG_A	*RTEL1*	c.597+3_597+80del		splice donor &splice donor 5th base &coding sequence &intron	.	.	.	.	.	.	.	LP
20_63685593_A_G	*RTEL1*	c.593A>G	p.Tyr198Cys	missense	.	29	.	deleterious (0.998)	0.63	0.808	.	LP
20_63685825_C_T	*RTEL1*	c.632C>T	p.Thr211Met	missense	0.000454	16.46	.	deleterious (0.629)	0.33	0.251	uncertain significance	VUS
20_63689531_GA_G	*RTEL1*	c.1241del	p.Asn414MetfsTer5	frameshift	.	.	.	.	.	.	.	P
20_63689787_C_G	*RTEL1*	c.1394C>G	p.Ser465Cys	missense	4.05E-06	26.8	.	deleterious (0.956)	0.50	0.77	uncertain significance	LP
20_63690206_G_A	*RTEL1*	c.1592G>A	p.Arg531Gln	missense	0.000152	19.86	.	deleterious (0.513)	0.37	0.055	uncertain significance	LP
20_63693211_C_T	*RTEL1*	c.2251C>T	p.Arg751Ter	stop gained	3.21E-05	39	.	.	.	.	pathogenic	P
20_63693247_C_T	*RTEL1*	c.2287C>T	p.Arg763Ter	stop gained	7.24E-05	36	.	.	.	.	pathogenic/ likely pathogenic&pathogenic	P
20_63694447_ACCAGGGCAGGCCCCACCTGTCGC_A	*RTEL1*	c.2405_2427del	p.Gly802AlafsTer34	frameshift	.	.	.	.	.	.	.	P
20_63694866_C_T	*RTEL1*	c.2566C>T	p.Leu856Phe	missense	8.05E-06	15.6	.	neutral (0.163)	0.49	0.187	.	VUS
10_79557237_T_C	*SFTPA2*	c.719A>G	p.Tyr240Cys	missense	.	22.5	0.3139	deleterious (0.816)	0.42	0.062	.	VUS
10_79560362_A_G	*SFTPA2*	c.-54+2T>C		splice donor	.	23.9	.	.	.	.	.	VUS
12_122474027_C_T	*ZCCHC8*	c.880G>A	p.Glu294Lys	missense	.	22.9	.	deleterious (0.528)	0.58	0.139	.	VUS
4_163128979_A_C	*NAF1*	c.1403T>G	p.Leu468Arg	missense	5.49E-06	19.09	0.2804	deleterious (0.636)	0.57	0.13	.	VUS

Supplementary Table 4. Qualifying variants identified in PROFILE.

Variant	Gene	HGVSp	HGVSp	Effect	GnomADg AF	CADD	Alpha Missense	Condel	Primate AI	REVEL	ClinVar Sig	ACMG Class
4_163129003_G_C	*NAF1*	c.1379C>G	p.Pro460Arg	missense	0.000129	19.87	0.158	deleterious (0.989)	0.68	0.059	.	VUS
4_163133221_T_G	*NAF1*	c.966A>C	p.Glu322Asp	missense	0.00043	24.2	0.3312	deleterious (0.940)	0.48	0.144	.	VUS
4_163133241_C_G	*NAF1*	c.946G>C	p.Asp316His	missense	.	27.1	0.9948	deleterious (1.000)	0.79	0.86	.	VUS
4_163164384_C_T	*NAF1*	c.373G>A	p.Asp125Asn	missense	0.000767	24.9	0.1694	deleterious (0.530)	0.84	0.145	.	VUS
4_163164392_C_T	*NAF1*	c.366-1G>A		splice acceptor	.	34	.	.	.	.	.	LP
4_163166459_G_A	*NAF1*	c.269C>T	p.Pro90Leu	missense	4.81E-05	16.81	0.081	deleterious (0.477)	0.56	0.027	.	VUS
5_169588441_C_T	*SPDL1*	c.25C>T	p.Leu9Phe	missense	.	25.2	0.3016	deleterious (1.000)	0.47	0.286	.	VUS
5_169588492_T_C	*SPDL1*	c.74+2T>C		splice donor &NMD transcript	3.58E-05	27.3	.	.	0.68	.	.	VUS
5_169588558_A_G	*SPDL1*	c.142A>G	p.Met48Val	missense	4.01E-06	23.2	0.162	deleterious (0.536)	0.53	0.091	.	VUS
5_169591177_C_T	*SPDL1*	c.289C>T	p.Gln97Ter	stop gained	.	35	.	.	.	.	.	LP
5_169591225_G_A	*SPDL1*	c.336+1G>A		splice donor	8.00E-06	33	.	.	.	.	.	VUS
5_169594221_G_T	*SPDL1*	c.608G>T	p.Arg203Leu	missense	0.000442	24.7	0.1523	deleterious (0.550)	0.24	0.063	.	VUS
5_169594287_C_T	*SPDL1*	c.674C>T	p.Ala225Val	missense	0.0002	23	0.2653	deleterious (0.999)	0.54	0.233	.	VUS
5_169596559_A_G	*SPDL1*	c.892-2A>G		splice acceptor	0.000469	35	.	.	.	.	likely benign	VUS
5_169599042_G_A	*SPDL1*	c.1207G>A	p.Ala403Thr	missense	.	21.1	0.1016	deleterious (0.479)	0.57	0.057	.	VUS
3_169764827_G_A	*TERC*	n.221C>T		non coding transcript exon	.	15.5	.	.	.	.	uncertain significance	VUS
3_169764881_G_A	*TERC*	n.167C>T		non coding transcript exon	.	22.5	.	.	.	.	pathogenic	VUS
3_169765024_T_A, C	*TERC*	n.24A>T		non coding transcript exon	1.30E-05	7.742	.	.	.	.	uncertain significance	LP

Supplementary Table 4. Qualifying variants identified in PROFILE.

Variant	Gene	HGVSp	HGVSp	Effect	GnomADg AF	CADD	Alpha Missense	Condel	Primate AI	REVEL	ClinVar Sig	ACMG Class
3_169765026_G_A	*TERC*	n.22C>T		non coding transcript exon	2.17E-05	2	.	.	.	.	uncertain significance	VUS

HGVS, Human Genome Variation Society; VUS, variant of uncertain significance; P, pathogenic; LP, likely pathogenic

Supplementary Table 5. Alternative definitions for qualifying variants and the rare synonymous used for sensitivity analyses.

	Ultra-rare PTV	Ultra-rare Ensemble# (PTV + Missense + Indel)	Rare PTV only	Rare Ensemble# (PTV + Missense + Indel)	Semi-rare PTV only	Semi-rare Ensemble# (PTV + Missense + Indel)	Rare synonymous^
Missense AF*	-	0	-	0.0005	-	0.01	-
PTV AF*	0	0	0.001	0.001	0.01	0.01	-
Consensus in silico prediction for missense:&							
Polyphen2 Humdiv	-	Probably	-	Probably	-	Probably	-
REVEL	-	>0.5	-	>0.5	-	>0.5	-
PrimateAI	-	>0.8	-	>0.8	-	>0.8	-
Variants (n)	13	30	28	67	29	78	38

*Below threshold for any population in gnomAD v2.1 exomes (AFR, AMR, ASJ, EAS, FIN, NFE, OTH, SAS) or gnomAD v3.2 genomes (AFR, AMR, ASJ, EAS, FIN, MID, NFE, OTH, SAS) or in The 1000 Genomes Project Phase 3 genomes (AFR, AMR, EAS, EUR, SAS).

&Consensus of three predictors (Polyphen2, REVEL, PrimateAI) for missense variants only if >2 out of 3, or 2 out of 2, or 1 out of 1 filters pass. Some predictors may have missing values.

^Allele frequency cutoff of 0.0005 in any population in gnomAD v2.1 exomes (AFR, AMR, ASJ, EAS, FIN, NFE, OTH, SAS) or gnomAD v3.2 genomes (AFR, AMR, ASJ, EAS, FIN, MID, NFE, OTH, SAS) or in The 1000 Genomes Project Phase 3 genomes (AFR, AMR, EAS, EUR, SAS), only synonymous variants.

#Ensemble models include non-coding *TERC* variants selected if passing missense AF level and involved in intramolecular base-pairing or previously described in pulmonary fibrosis or dyskeratosis congenita or hoyeraal hreidarsson.

PTV: Protein truncating variants.

Supplementary Table 6. Common IPF risk variants and effects considered for PRS-IPF estimation.

Locus	SNP ID	Chr.	Position (hg38)	Effect	Non-effect	OR	p-value
KIF15	rs141979279	3	44,816,639	C	T	1.50	$1.21x10^{-10}$
TERC	rs10936601	3	169,810,661	C	T	0.79	$2.10x10^{-15}$
FAM13A	rs2013701	4	88,963,935	G	T	1.25	$4.60x10^{-16}$
TERT	rs7725218	5	1,282,299	G	A	1.41	$4.90x10^{-32}$
DSP	rs2076295	6	7,562,999	G	T	1.49	$1.50x10^{-48}$
MAD1L1	rs12699415	7	1,869,843	A	G	1.27	$7.85x10^{-18}$
ZKSCAN1	rs2897075	7	100,032,719	T	C	1.30	$1.77x10^{-21}$
DEPTOR	rs28513081	8	119,921,886	A	G	1.20	$1.22x10^{-9}$
10q25.1	rs79684490	10	109,470,103	A	G	1.40	$3.52x10^{-8}$
MUC5B	rs35705950	11	1,219,991	T	G	5.06	$9.09x10^{-418}$
ATP11A	rs12585036	13	112,881,427	C	T	1.29	$5.99x10^{-14}$
IVD	rs59424629	15	40,428,343	T	G	1.27	$4.98x10^{-19}$
KNL1	rs12912339	15	40,639,510	A	G	1.30	$7.41x10^{-13}$
AKAP13	rs62023891	15	85,553,985	A	G	1.18	$1.32x10^{-8}$
NPRL3	rs74614704	16	112,241	A	G	1.49	$2.57x10^{-12}$
17q21.31	rs3785884	17	45,980,229	G	A	1.40	$2.53x10^{-20}$
DPP9	rs35574495	19	4,686,976	G	T	0.80	$1.08x10^{-9}$
STMN3	rs112087793	20	63,652,817	C	T	1.34	$1.09x10^{-8}$
RTEL1	rs41308092	20	63,693,038	A	G	1.75	$3.13x10^{-9}$

SNP: single nucleotide polymorphism; Chr.: chromosome; OR: odds ratio; p-value: significance in the original study

Supplementary Table 7. Common telomere length variants and effects considered for PRS-TL estimation.

Locus	SNP ID	Chr.	Position (hg38)	Effect	Non-effect	Beta	p-value
PARP1	rs3219104	1	226374920	C	A	0.042	$9.60x10^{-11}$
TERC	rs10936600	3	169796797	T	A	-0.086	$7.18x10^{-51}$
NAF1	rs4691895	4	163127047	C	G	0.058	$1.58x10^{-21}$
TERT	rs7705526	5	1285859	A	C	0.082	$5.34x10^{-45}$
TERT	rs2853677	5	1287079	A	G	-0.064	$3.35x10^{-31}$
POT1	rs59294613	7	124914213	A	C	-0.041	$1.17x10^{-13}$
STN1	rs9419958	10	103916188	C	T	-0.064	$5.05x10^{-19}$
ATM	rs228595	11	108234866	A	G	-0.029	$1.43x10^{-8}$
DCAF4	rs2302588	14	72938044	C	G	0.048	$1.68x10^{-8}$
MPHOSPH6	rs7194734	16	82166375	T	C	-0.037	$6.94x10^{-10}$
ZNF208	rs8105767	19	22032639	G	A	0.039	$5.42x10^{-13}$
RTEL1/ STMN3	rs75691080	20	63638397	T	C	-0.067	$5.99x10^{-14}$
RTEL1	rs34978822	20	63660246	G	C	-0.140	$7.26x10^{-10}$
RTEL1/ ZBTB46	rs73624724	20	63805045	C	T	0.051	$6.33x10^{-12}$
SENP7	Rs551442	3	101346524	T	C	-0.037	$2.45x10^{-8}$
MOB1B	rs13137667	4	70908630	C	T	0.077	$2.43x10^{-8}$
CARMIL1	rs34991172	6	25480100	G	T	-0.061	$6.19x10^{-9}$
PRRC2A	rs2736176	6	31619784	C	G	0.035	$3.53x10^{-10}$
TERF2	rs3785074	16	69373083	G	A	0.035	$4.64x10^{-10}$
RFWD3	rs62053580	16	74646176	G	A	-0.039	$4.08x10^{-8}$

SNP: single nucleotide polymorphism; Chr.: chromosome; Beta: effect; p-value: significance in the original study

Supplementary Table 8. Schoenfeld residuals test for proportionality assumption of the Cox models.

		PFFPR	PROFILE
Model	Variable	p-value	p-value
1	**All variants**	0.371	0.663
	Age	0.348	0.361
	Sex	0.947	0.386
	PC1	0.433	0.085
	PC2	0.660	0.793
	MUC5B	0.356	0.890
	Smoking	0.051	0.423
	FVC pred	0.646	0.011
	DLCO pred	0.587	0.013
	Global	0.313	0.054
2	**Pathogenic variants**	0.817	0.709
	Age	0.349	0.300
	Sex	0.990	0.388
	PC1	0.418	0.065
	PC2	0.629	0.820
	MUC5B	0.338	0.815
	Smoking	0.052	0.439
	FVC pred	0.616	0.012
	DLCO pred	0.559	0.023
	Global	0.344	0.059
3	**Telomeric**	0.195	0.768
	Age	0.350	0.328
	Sex	0.953	0.392
	PC1	0.427	0.079
	PC2	0.635	0.806
	MUC5B	0.339	0.956
	Smoking	0.049	0.411
	FVC pred	0.645	0.012
	DLCO pred	0.587	0.016
	Global	0.236	0.058
4	**Pathogenic telomeric variants**	0.945	0.331
	Age	0.350	0.277
	Sex	0.987	0.429
	PC1	0.418	0.064
	PC2	0.628	0.790
	MUC5B	0.337	0.885
	Smoking	0.052	0.409
	FVC pred	0.617	0.012
	DLCO pred	0.559	0.021
	Global	0.354	0.038
5	**Non-telomere variants**	0.646	0.973
	Age	0.340	0.331
	Sex	0.970	0.361
	PC1	0.438	0.079
	PC2	0.654	0.722
	MUC5B	0.335	0.954
	Smoking	0.051	0.355
	FVC pred	0.624	0.011
	DLCO pred	0.567	0.014
	Global	0.324	0.049
6	**Surfactant variants**	0.554	0.986
	Age	0.341	0.343
	Sex	0.987	0.365
	PC1	0.439	0.081
	PC2	0.687	0.730
	MUC5B	0.328	0.927
	Smoking	0.049	0.370
	FVC pred	0.606	0.010
	DLCO pred	0.547	0.013
	Global	0.308	0.050
7	***RTEL1/TERT/PARN* variants**	0.351	0.989
	Age	0.345	0.290
	Sex	0.982	0.424
	PC1	0.415	0.074
	PC2	0.625	0.767
	MUC5B	0.341	0.975
	Smoking	0.047	0.438

	FVC pred	0.630	0.012
	DLCO pred	0.573	0.016
	Global	0.249	0.057
**8**	***RTEL1* variants**	0.280	0.466
	Age	0.350	0.341
	Sex	0.98	0.417
	PC1	0.42	0.072
	PC2	0.62	0.770
	MUC5B	0.33	0.984
	Smoking	0.05	0.389
	FVC pred	0.63	0.010
	DLCO pred	0.57	0.015
	Global	0.250	0.040
**9**	***TERT* variants**	0.665	0.119
	Age	0.342	0.309
	Sex	0.975	0.463
	PC1	0.430	0.073
	PC2	0.632	0.701
	MUC5B	0.322	0.797
	Smoking	0.049	0.358
	FVC pred	0.625	0.009
	DLCO pred	0.568	0.012
	Global	0.329	0.032
**10**	***PARN* variants**	0.870	0.497
	Age	0.348	0.341
	Sex	0.988	0.360
	PC1	0.423	0.082
	PC2	0.624	0.730
	MUC5B	0.338	0.925
	Smoking	0.048	0.373
	FVC pred	0.628	0.011
	DLCO pred	0.571	0.013
	Global	0.338	0.046
**11**	***SPDL1* variants**	0.670	0.753
	Age	0.342	0.296
	Sex	0.978	0.358
	PC1	0.436	0.076
	PC2	0.621	0.730
	MUC5B	0.327	0.954
	Smoking	0.051	0.366
	FVC pred	0.626	0.011
	DLCO pred	0.571	0.016
	Global	0.330	0.048
**12**	***KIF15* variants**	0.800	0.610
	Age	0.340	0.348
	Sex	0.970	0.359
	PC1	0.430	0.085
	PC2	0.630	0.774
	MUC5B	0.320	0.895
	Smoking	0.050	0.405
	FVC pred	0.630	0.011
	DLCO pred	0.570	0.015
	Global	0.350	0.057
**13**	**ClinVar variants**	0.797	0.059
	Age	0.342	0.242
	Sex	0.964	0.440
	PC1	0.424	0.065
	PC2	0.631	0.704
	MUC5B	0.335	0.791
	Smoking	0.049	0.436
	FVC pred	0.632	0.009
	DLCO pred	0.575	0.013
	Global	0.344	0.021
**14**	**PRS-IPF tertile 1**	0.990	0.989
	Age	0.340	0.341
	Sex	0.980	0.358
	PC1	0.430	0.081
	PC2	0.630	0.733
	MUC5B	0.320	0.932
	Smoking	0.050	0.369
	FVC pred	0.630	0.011
	DLCO pred	0.570	0.013
	Global	0.180	0.050
**15**	**PRS-IPF tertile 2**	0.590	0.899
	Age	0.340	0.316
	Sex	0.980	0.346

	PC1	0.430	0.075
	PC2	0.630	0.728
	MUC5B	0.320	0.950
	Smoking	0.050	0.391
	FVC pred	0.630	0.011
	DLCO pred	0.570	0.012
	Global	0.280	0.017
**16**	**PRS-IPF tertile 3**	0.570	0.824
	Age	0.340	0.356
	Sex	0.980	0.410
	PC1	0.430	0.071
	PC2	0.630	0.699
	MUC5B	0.320	0.918
	Smoking	0.050	0.402
	FVC pred	0.630	0.010
	DLCO pred	0.570	0.014
	Global	0.350	0.037

FVC, forced vital capacity; DLCO, predicted diffusing capacity of the lungs for monoxide; PC: principal component

Supplementary Table 9. Genomic predictors of whole-genome sequencing telomere length (WGS-TL) in multivariate linear regression model stratified by groups of carriers with qualifying variants.

	All variants			Telomere variants			Non-telomere variants		
	**Beta**	**95% CI**	**p-value**	**Beta**	**95% CI**	**p-value**	**Beta**	**95% CI**	**p-value**
**Carrier**	-0.243	-0.377, -0.108	4.21×10^{-4}	-0.360	-0.513, -0.206	4.86×10^{-6}	0.099	-0.143, 0.340	0.423
**Age**	-0.002	-0.009, 0.004	0.495	-0.003	-0.009, 0.004	0.385	0.000	-0.007, 0.006	0.930
**Male sex**	-0.011	-0.126, 0.104	0.495	-0.022	-0.136, 0.093	0.713	0.001	-0.114, 0.117	0.985
**PC1**	1.21	-0.255, 2.67	0.105	1.16	-0.292, 2.62	0.117	1.09	-0.386, 2.56	0.148
**PC2**	-0.683	-2.16, 0.793	0.364	-0.458	-1.93, 1.02	0.542	-0.672	-2.16, 0.821	0.377

PC: principal component; CI: confidence interval.

Supplementary Table 10. Results of the random effects models with the age at diagnosis as the frailty term.

	PFFPR	PROFILE
	**HR (95% CI), p-value**	**HR (95% CI), p-value**
**All variants**	1.61 (1.04-2.50), p=0.03	1.37 (0.99-1.90), p=0.06
**Pathogenic**	1.77 (0.96-3.26), p=0.07	1.97 (1.27-3.04), p=2.40x10^{-3}
**Telomeric**	1.54 (0.95-2.50), p=0.08	1.65 (1.14-2.38), p=7.30x10^{-3}
**Pathogenic telomeric**	1.90 (1.00-3.63), p=0.05	2.01 (1.27-3.20), p=3.10x10^{-3}
**Non-telomere**	1.67 (0.70-4.02), p=0.25	0.85 (0.45-157), p=0.60
**Surfactant**	2.09 (0.45-9.72), p=0.35	1.04 (0.95-3.35), p=0.94
**ClinVar**	1.42 (0.75-2.70), p=0.28	2.49 (1.63-3.83), p=2.80x10^{-5}
TERT/PARN/RTEL1	1.47 (0.85-2.55), p=0.17	1.76 (1.17-2.65), p=6.30x10^{-3}
RTEL1	1.34 (0.61-2.96), p=0.47	1.51 (0.82-2.81), p=0.19
TERT	1.01 (0.40-2.55), p=0.98	3.53 (1.83-6.79), p=1.60x10^{-4}
PARN	2.82 (0.90-8.84), p=0.07	1.07 (0.49-2.32), p=0.87
SPDL1	1.98 (0.38-10.25), p=0.41	0.60 (0.24-1.49), p=0.27
KIF15	1.22 (0.33-4.47), p=0.77	1.54 (0.49-4.87), p=0.46
MUC5B	0.44 (0.31-0.64), p=1.50x10^{-5}	0.59 (0.45-0.76), p=6.20x10^{-5}
**PRS (Tertile 1)**	1.82 (1.28-2.60), p=9.50x10^{-4}	1.50 (1.15-1.95), p=2.90x10^{-3}
**PRS (Tertile 2)**	0.74 (0.49-1.11), p=0.15	1.16 (0.89-1.51), p=0.28
**PRS (Tertile 3)**	0.66 (0.42-1.05), p=0.08	0.59 (0.45-0.78), p=1.90x10^{-4}

HR: hazard ratio; CI: confidence interval

Supplementary Table 11. Results of the random effects models with the predicted FVC as the frailty term.

	PFFPR	PROFILE
	**HR (95% CI), p-value**	**HR (95% CI), p-value**
**All variants**	1.53 (1.12-2.10), $p=7.20x10^{-3}$	1.67 (1.01-2.78), p=0.04
**Pathogenic**	1.75 (1.13-2.70), p=0.01	2.19 (1.17-4.11), p=0.01
**Telomeric**	1.50 (1.06-2.13), p=0.02	2.00 (1.14-3.53), p=0.02
**Pathogenic telomeric**	1.80 (1.16-2.81), $p=9.30x10^{-3}$	2.60 (1.32-5.10), $p=5.20x10^{-3}$
**Non-telomere**	1.41 (0.80-2.48), p=0.24	0.90 (0.36-2.24), p=0.83
**Surfactant**	1.71 (0.62-4.73), p=0.29	2.02 (0.32-12.88), p=0.45
**ClinVar**	1.38 (0.89-2.13), p=0.15	3.11 (1.63-5.95), $p=6.10x10^{-4}$
TERT/PARN/RTEL1	1.48 (1.00-2.18), p=0.05	2.14 (1.16-3.96), p=0.02
RTEL1	1.40 (0.80-2.46), p=0.24	2.14 (0.78-5.90), p=0.14
TERT	1.09 (0.57-2.07), p=0.79	4.48 (1.66-1204), $p=3.00x10^{-3}$
PARN	2.38 (1.17-4.87), p=0.017	0.88 (0.31-2.47), p=0.81
SPDL1	1.30 (0.48-3.53), p=0.61	0.58 (0.15-2.16), p=0.42
KIF15	1.27 (0.52-3.11), p=0.60	0.98 (0.19-5.13), p=0.98
MUC5B	0.54 (0.42-0.70), $p=2.20x10^{-6}$	0.52 (0.35-0.76), $p=6.00x10^{-4}$
**PRS (Tertile 1)**	1.61 (1.26-2.08), $p=1.70x10^{-4}$	1.82 (1.23-2.71), $p=3.00x10^{-3}$
**PRS (Tertile 2)**	0.76 (0.58-0.99), p=0.04	1.10 (0.75-1.60), p=0.61
**PRS (Tertile 3)**	0.79 (1.25-0.61), p=0.10	0.50 (0.34-0.75), $p=7.30x10^{-4}$

FVC, forced vital capacity; HR: hazard ratio; CI: confidence interval.

Supplementary Table 12. Results of the random effects models with the predicted DLCO as the frailty term.

	PFFPR	PROFILE
	**HR (95% CI), p-value**	**HR (95% CI), p-value**
**All variants**	1.53 (1.12-2.10), p=7.20x10^{-3}	1.69 (1.06-2.72), p=0.03
**Pathogenic**	1.71 (1.10-2.65), p=0.02	2.29 (1.20-4.35), p=0.01
**Telomeric**	1.49 (1.05-2.12), p=0.03	1.94 (1.13-3.32), p=0.02
**Pathogenic telomeric**	1.77 (1.13-2.76), p=0.01	2.33 (1.15-4.70), p=0.02
**Non-telomere**	1.45 (0.83-2.57), p=0.19	1.04 (0.43-2.53), p=0.91
**Surfactant**	1.83 (0.66-5.07), p=0.25	0.78 (0.14-4.21), p=0.77
**ClinVar**	1.36 (0.88-2.10), p=0.15	2.46 (1.37-4.41), p=2.30x10^{-3}
TERT/PARN/RTEL1	1.46 (0.99-2.16), p=0.06	1.95 (1.06-3.55), p=0.03
RTEL1	1.40 (0.80-2.47), p=0.24	2.61 (0.91-7.50), p=0.07
TERT	1.08 (0.57-2.06), p=0.81	3.11 (1.23-7.89), p=0.02
PARN	2.25 (1.09-4.64), p=0.03	0.79 (0.27-2.27), p=0.66
SPDL1	1.30 (0.48-3.51), p=0.61	0.95 (0.26-3.58), p=0.95
KIF15	1.27 (0.52-3.10), p=0.60	1.54 (0.34-7.01), p=0.58
MUC5B	0.54 (0.42-0.70), p=2.70x10^{-6}	0.54 (0.37-0.79), p=1.30x10^{-3}
**PRS (Tertile 1)**	1.61 (1.26-2.07), p=1.80x10^{4}	1.90 (1.27-2.87), p=1.90x10^{-3}
**PRS (Tertile 2)**	0.76 (0.58-0.99), p=0.05	1.18 (0.80-1.77), p=0.40
**PRS (Tertile 3)**	0.79 (0.60-1.04), p=0.09	0.43 (0.29-6.555), p=7.20x10^{-5}

DLCO, diffusing capacity of the lungs for carbon monoxide; HR: hazard ratio; CI: confidence interval.

Supplementary Table 13. Weighted Cox regression analysis in patients carrying rare qualifying variants in PROFILE.

	PROFILE
	**HR (95% CI), p-value**
**All variants**	1.39 (0.98-1.98), p=0.061
**Pathogenic**	2.03 (1.37-3.00), $p=4.40x10^{-4}$
**Telomeric**	1.69 (1.17-2.46), $p=5.57x10^{-3}$
**Pathogenic telomeric**	2.14 (1.38-3.33), $p=6.63x10^{-4}$
**Non-telomere**	0.83 (0.41-1.70), p=0.62
**Surfactant**	1.03 (0.23-4.63), p=0.96
**ClinVar**	2.36 (1.55-3.60), $p=6.30x10^{-5}$
TERT/PARN/RTEL1	1.79 (1.19-2.68), $p=4.99x10^{-3}$
RTEL1	1.62 (0.80-3.26), p=0.17
TERT	3.28 (1.96-5.47), $p=5.87x10^{-6}$
PARN	1.11 (0.58-2.15), p=0.75
SPDL1	0.60 (0.22-1.65), p=0.32
KIF15	1.49 (0.75-2.96), p=0.26
MUC5B	0.58 (0.44-0.76), $p=8.39x10^{-5}$
**PRS (Tertile 1)**	1.54 (1.16-2.03), $p=2.98x10^{-3}$
**PRS (Tertile 2)**	1.17 (0.89-1.55), p=0.26
**PRS (Tertile 3)**	0.57 (0.42-0.76), $p=1.77x10^{-4}$

HR: hazard ratio; CI: Confidence interval.

Supplementary Table 14. Cox regression analysis at 24, 36, 48 and 60 months (m) from diagnosis in patients carrying rare qualifying variants in PROFILE.

	24 m		36 m		48 m		60 m	
	**HR (95%CI)**	**p-value**	**HR (95%CI)**	**p-value**	**HR (95%CI)**	**p-value**	**HR (95%CI)**	**p-value**
**All variants**	1.52 (0.98-2.38)	0.064	1.40 (0.96-2.06)	0.082	1.40 (0.99-1.97)	0.058	1.37 (0.99-1.89)	0.058
**Pathogenic**	2.30 (1.33-3.98)	2.93×10^{-3}	1.94 (1.17-3.20)	9.76×10^{-3}	1.99 (1.26-3.14)	3.04×10^{-3}	1.98 (1.28-3.05)	2.13×10^{-3}
**Telomeric**	1.80 (1.09-2.97)	0.023	1.59 (1.04-2.45)	0.034	1.69 (1.15-2.48)	7.05×10^{-3}	1.66 (1.15-2.38)	6.60×10^{-3}
**ClinVar**	2.36 (1.27-4.37)	6.62×10^{-3}	1.99 (1.18-3.37)	0.02	2.16 (1.36-3.44)	1.10×10^{-3}	2.50 (1.63-3.83)	2.70×10^{-5}

HR: hazard ratio; CI: Confidence interval.

Supplementary Table 15. Cox regression analysis at 60 months (m) from diagnosis in non-transplant patients carrying rare qualifying variants in PFFPR.

	60 m	
	**HR (95%CI)**	**p-value**
**All variants (N=115)**	1.59 (1.08-2.35)	0.020
**Pathogenic (N=47)**	1.90 (1.11-3.26)	0.020
**Telomeric (N=82)**	1.55 (1.01-2.39)	0.047
**ClinVar (N=47)**	0.95 (0.50-1.81)	0.871

HR: hazard ratio; CI: Confidence interval.

Supplementary Figures

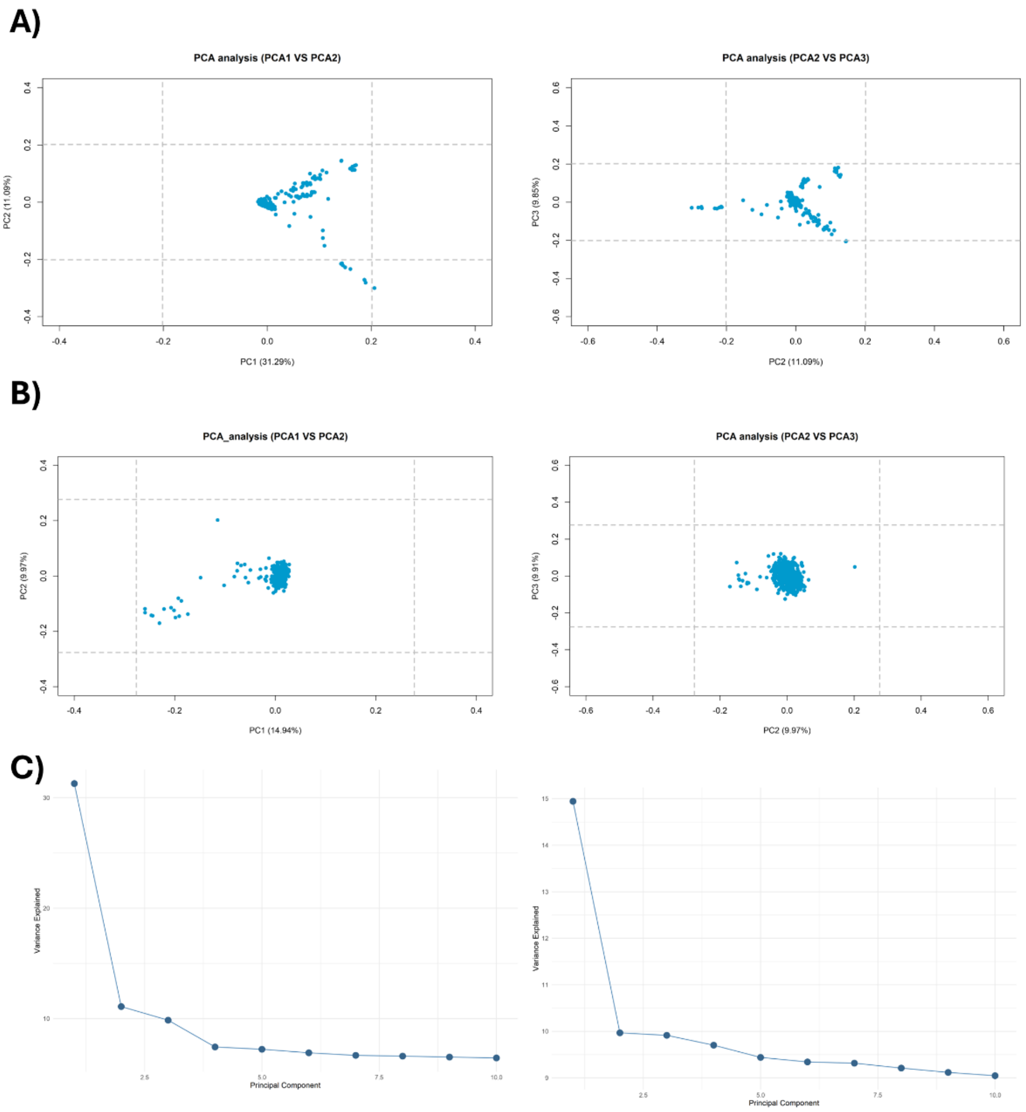

Supplementary Figure 1. Principal component analysis. A) Plot of the first two (left) and the second and third (right) principal components of genetic variation of IPF patients in the PFFPR. B) Plot of the first two (left) and the second and third (right) principal components of genetic variation of IPF patient in PROFILE. C) Proportion of variance explained by each PC (PFFPR on the right, and PROFILE on the left).

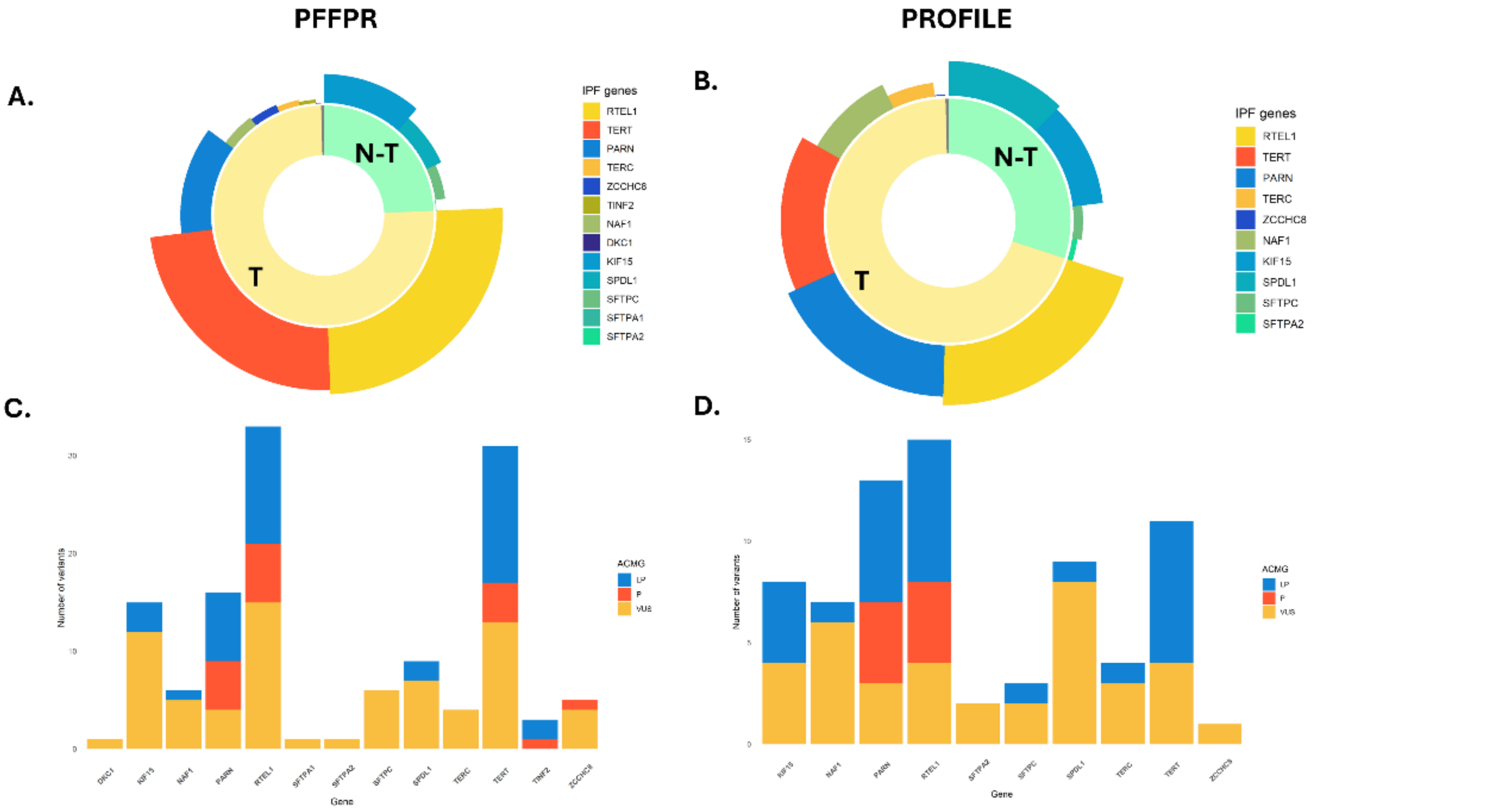

Supplementary Figure 2. Distribution of qualifying variants (QV) in monogenic adult-onset pulmonary fibrosis (PF) genes in the PFFPF and PROFILE cohorts. A) Total QVs in monogenic adult-onset PF genes in the PFFPR. B) Total QVs in monogenic adult-onset PF genes in the PROFILE cohort. C) Variants classified in P/LP/VUS per gene in the PFFPR. D) Variants classified in P/LP/VUS per gene in the PROFILE cohort. T: Telomere; N-T: Non-telomere.

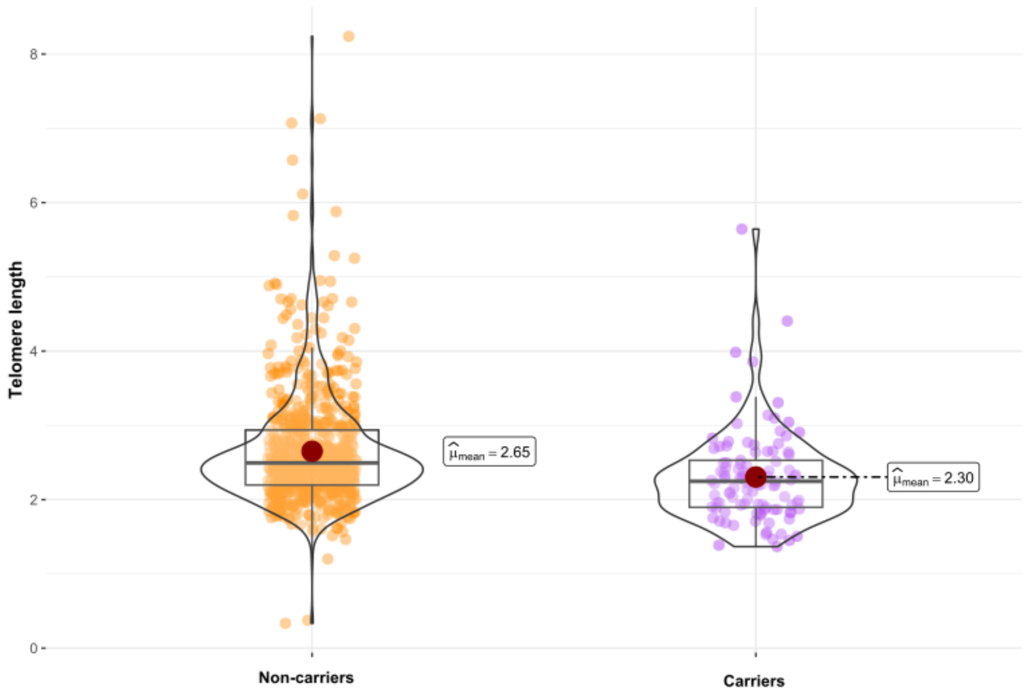

Supplementary Figure 3. Violin plots comparing telomere length estimated by whole-genome sequencing (WGS-TL) between carriers of qualifying variants in telomere genes and non-carriers.

A.

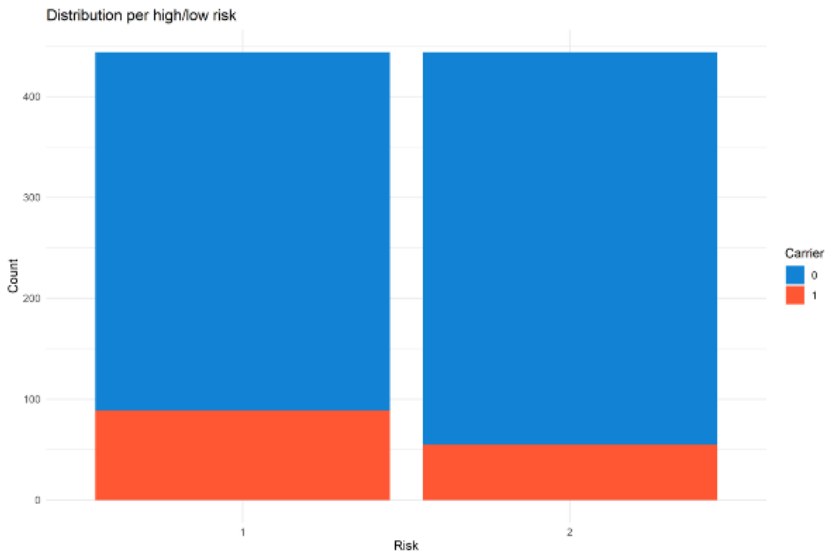

B.

	Low risk	High risk
OR (95% CI)	1.74 (1.20-2.53)	reference
p-value	3.57×10^{-3}	reference

Supplementary Figure 4. Association between prevalence of qualifying variants (QV) and PRS-IPF in the PFFPR. A) Distribution of carriers (1) and non-carriers (0) in low and high PRS-IPF. B) Risk of carrying a QV in patients with low polygenic risk in comparison with individuals with high polygenic risk. The odds ratio (OR) and the 95% confidence interval (CI) were estimated using logistic regression adjusted by age of diagnosis, sex, and the two main principal components.

A.

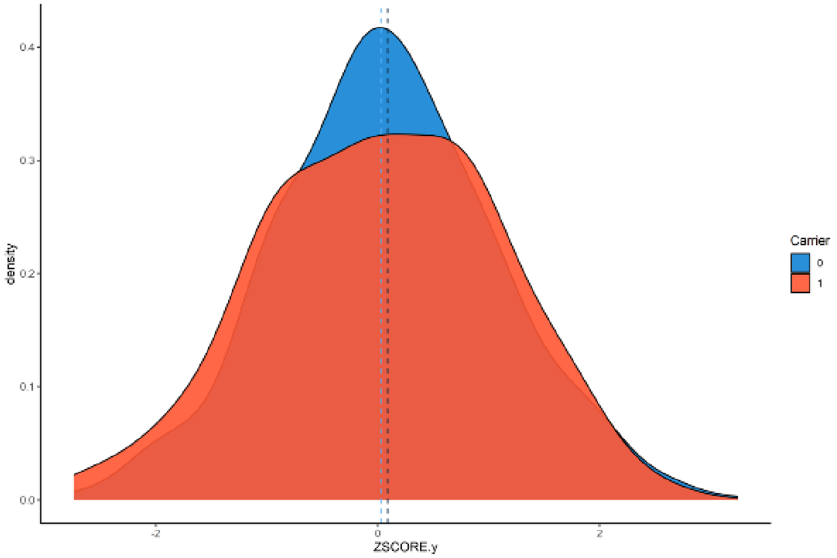

B.

	T1	T2	T3
**OR (95% CI)**	1.60 (1.01-2.54)	Reference	1.46 (0.92-2.32)
**p-value**	0.05	Reference	0.11

Supplementary Figure 5. Association between prevalence of qualifying variants (QV) and PRS-IPF (after excluding the *MUC5B* locus) in the PFFPR. A) Distribution of PRS-IPF in carriers (1) and non-carriers (0). Vertical dotted lines represent the mean value of the distribution. B) Risk of carrying a QV for patients with low polygenic risk (T1) and high polygenic risk (T3) compared to those in the middle tertile. The odds ratios (OR) and the 95% confidence intervals (CI) were estimated using logistic regression adjusted for age of diagnosis, sex, and the two main principal components.

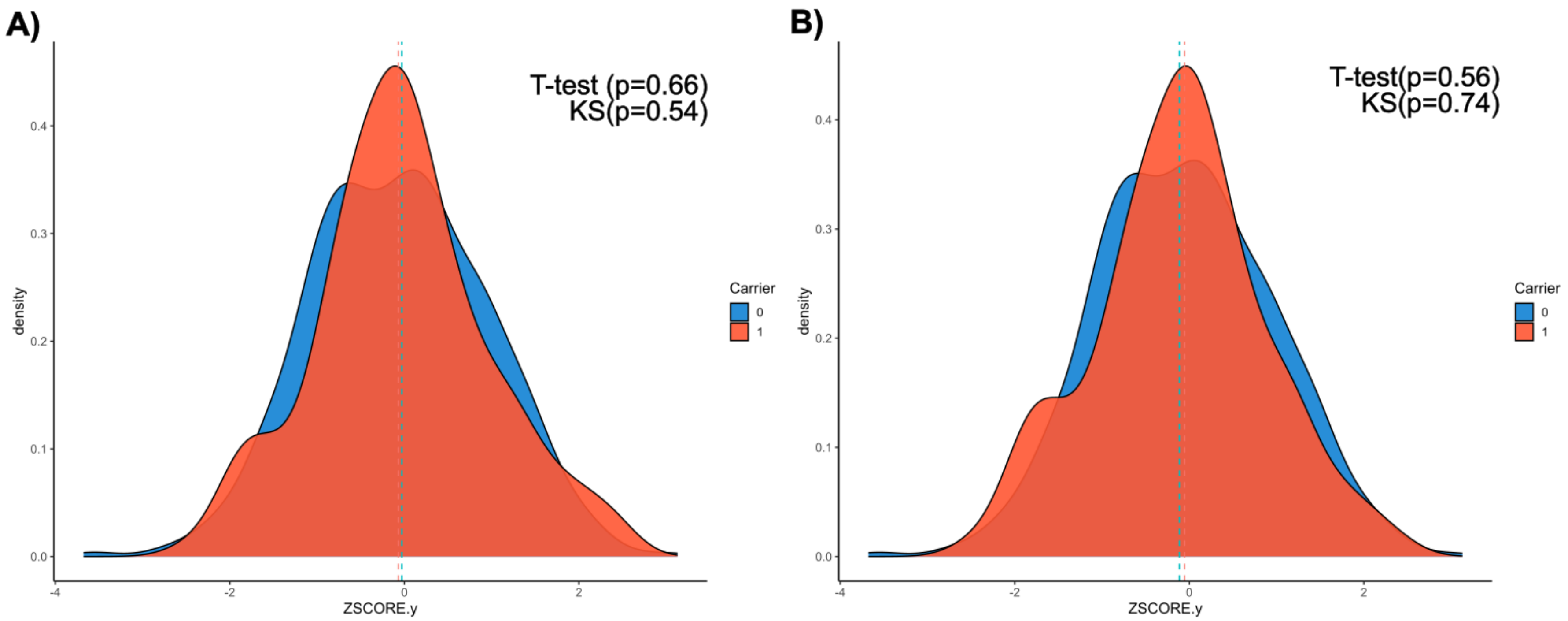

Supplementary Figure 6. Association between the prevalence of qualifying variants (QV) and PRS-TL in the PFFPR. Distribution of PRS-TL in carriers (1) and non-carriers (0). Vertical dotted lines represent the mean value of the distribution A) Carriers (1) and non-carriers (0) in telomere and non-telomere genes. B) Carriers (1) and non-carriers (0) in telomere genes. T-test: Student's t-test; KS: Kolmogorov-Smirnov test.

A)

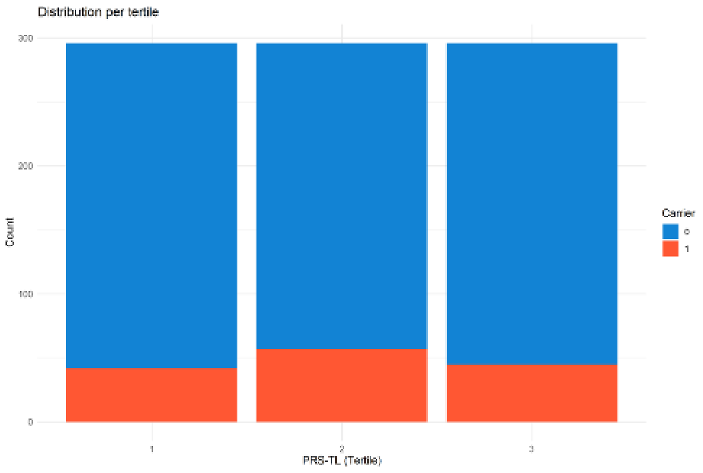

C)

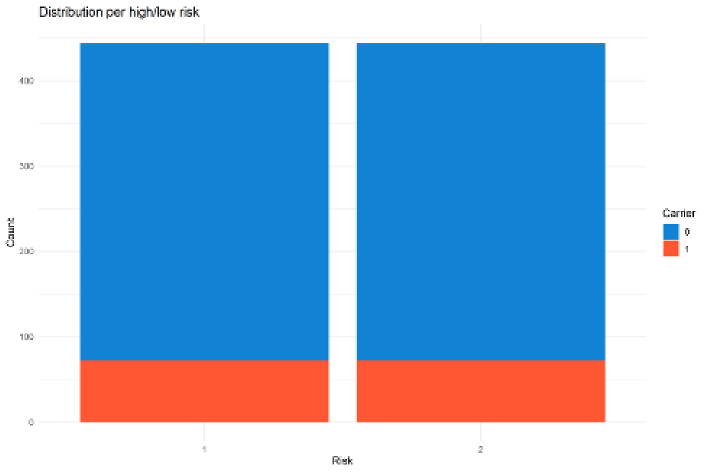

B)

	T1	T2	T3
OR (95% CI)	0.71 (0.46-1.12)	Reference	0.81 (0.53-1.26)
p-value	0.14	Reference	0.36

D)

	Low risk	High risk
OR (95% CI)	Reference	1.01 (0.70-1.46)
p-value	Reference	0.95

Supplementary Figure 7. Association between prevalence of qualifying variants (QV) in telomere and non-telomere genes and PRS-TL in the PFFPR. A) Distribution of carriers (1) and non-carriers (0) in PRS-TL tertiles. B) Risk of carrying a QV for individuals with low polygenic risk (T1) and high polygenic risk (T3) compared to those in the middle tertile. C) Distribution of carriers (1) and non-carriers (0) in high and low PRS-TL. D) Risk of carrying a QV in patients with high polygenic risk in comparison with patients with low polygenic risk. The odds ratios (OR) and the 95% confidence intervals (CI) were estimated using logistic regression adjusted by age of diagnosis, sex, and the two main principal components.

A)

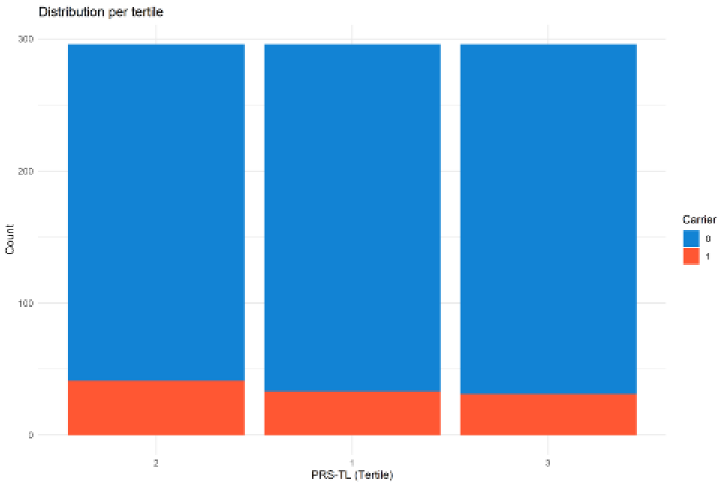

C)

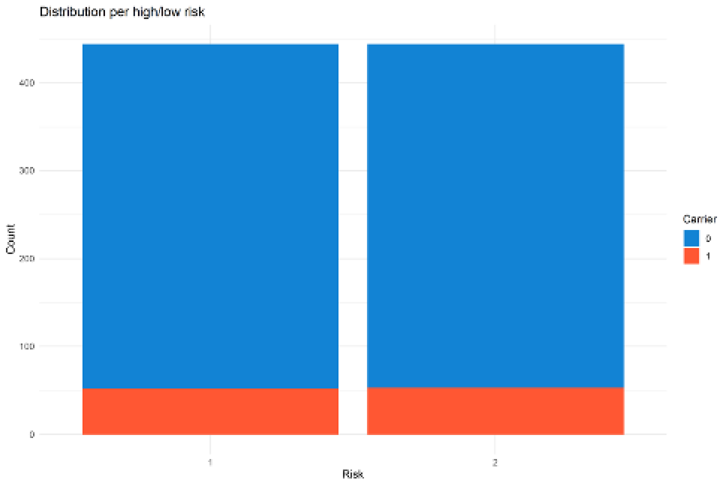

B)

	T1	T2	T3
**OR (95% CI)**	0.81 (0.49-1.34)	Reference	0.81 (0.49-1.35)
**p-value**	0.41	Reference	0.42

D)

	Low risk	High risk
**OR (95% CI)**	Reference	1.04 (0.69-1.59)
**p-value**	Reference	0.83

Supplementary Figure 8. Association between prevalence of qualifying variants (QV) in telomere genes and PRS-TL in the PFFPR. A) Distribution of carriers (1) and non-carriers (0) in PRS-TL tertiles. B) Risk of carrying a QV for individuals with low polygenic risk (T1) and high polygenic risk (T3) compared to those in the middle tertile. C) Distribution of carriers (1) and non-carriers (0) in high and low PRS-TL. D) Risk of carrying a QV in individuals with high polygenic risk in comparison with individuals with low polygenic risk. The odds ratios (OR) and the 95% confidence intervals (CI) were estimated using logistic regression adjusted by age of diagnosis, sex, and the two main principal components.

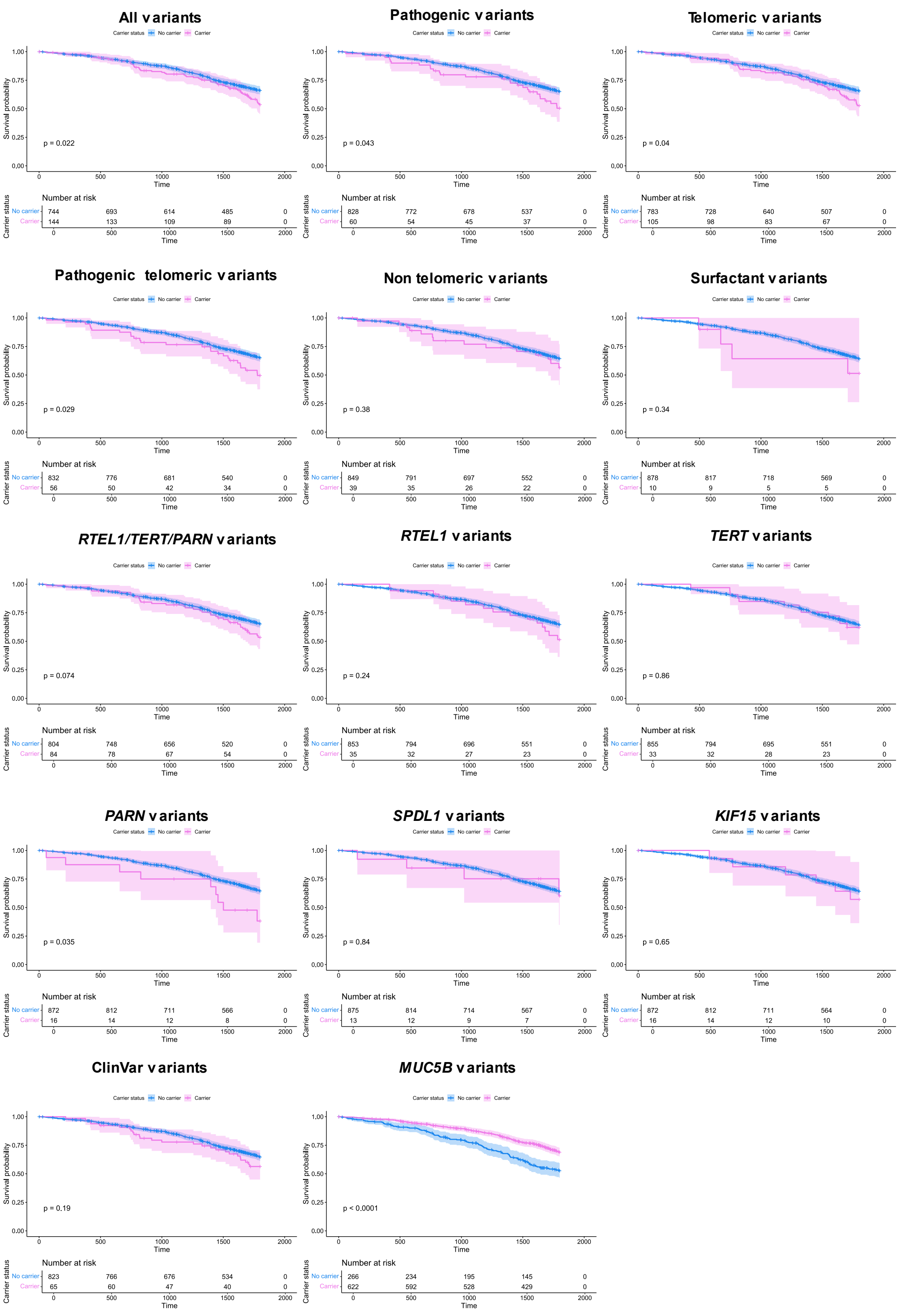

Supplementary Figure 9. Kaplan-Meier survival analysis for qualifying variants (QV) (per gene and group of genes) and the *MUC5B* risk allele in the PFFPR. p-values for the log-rank test are shown.

Variable	Carrier	Non carrier	HR, 95% CI, p-value
All variants	128	744	1.46 (1.04-2.03, p=0.027)
Pathogenic variants	48	824	1.46 (0.88-2.43, p=0.140)
Telomeric variants	89	783	1.37 (0.93-2.00, p=0.107)
Pathogenic telomeric variants	44	828	1.50 (0.89-2.52, p=0.128)
Clinvar	58	814	1.22 (0.76-1.96, p=0.416)
TERT/RTEL1 variants	68	804	1.30 (0.84-2.02, p=0.243)

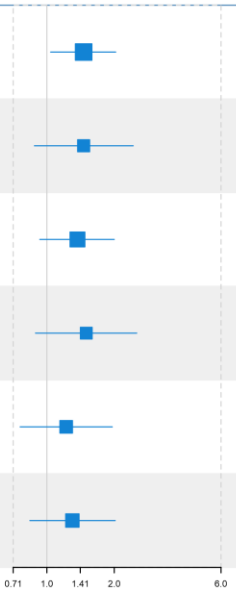

Supplementary Figure 10. Qualifying variants (QV) effect on survival in the PFFPR (excluding carriers of QV within *PARN*). All analysis were performed using Cox regression models adjusted for sex, age of diagnosis, the two main principal components, *MUC5B* risk allele, smoking history, forced vital capacity (FVC) % predicted, and diffusing capacity for carbon monoxide (DLCO) % predicted. The X-axis shows Hazard-ratios (HR); the grey solid line corresponds to the HR=1.0. The boxes correspond to adjusted HR and horizontal lines correspond to 95% confidence intervals (CI).

Variable	Carrier	Non carrier	HR, 95% CI, p-value
All rare variants	144	744	1.53 (1.12-2.10, p=7.33x10^{-3})
rare ensemble	64	824	1.34 (0.86-2.10, p=0.192)
rare PTV	28	860	1.36 (0.71-2.61, p=0.351)
semi ensemble	75	813	1.66 (1.11-2.50, p=0.014)
semi PTV	29	859	1.51 (0.81-2.82, p=0.194)
ultra ensemble	30	858	1.72 (0.98-3.02, p=0.057)
ultra PTV	13	875	2.44 (1.14-5.24, p=0.022)

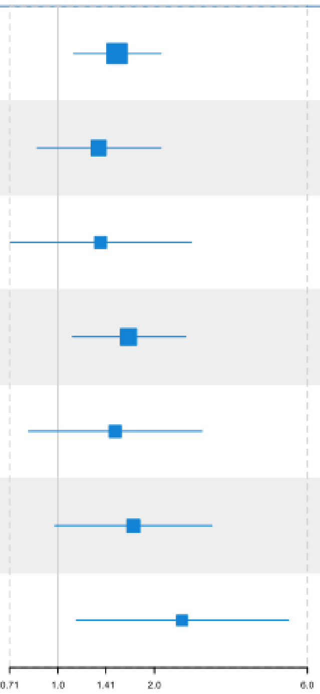

Supplementary Figure 11. **Alternative qualifying variants (QV) classifications and effects on survival in the PFFPR**. All analysis were performed using Cox regression models adjusted for sex, age of diagnosis, the two main principal components, *MUC5B* risk allele, smoking history, forced vital capacity (FVC) % predicted, and diffusing capacity for carbon monoxide (DLCO) % predicted. The X-axis shows Hazard-ratios (HR); the grey solid line corresponds to the HR=1.0. The boxes correspond to adjusted HR and horizontal lines correspond to 95% confidence intervals (CI).

A)

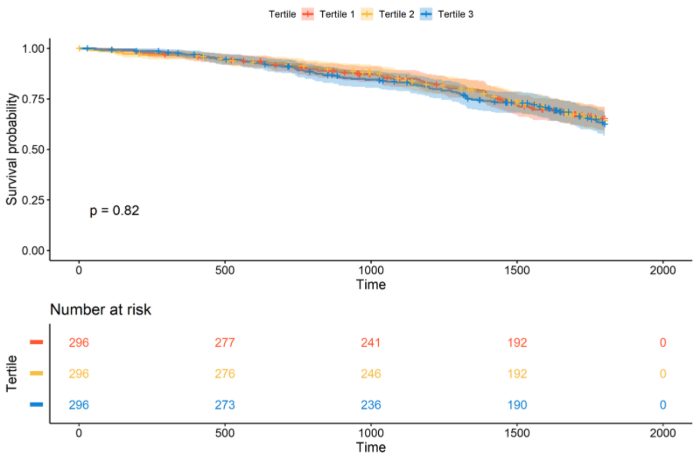

B)

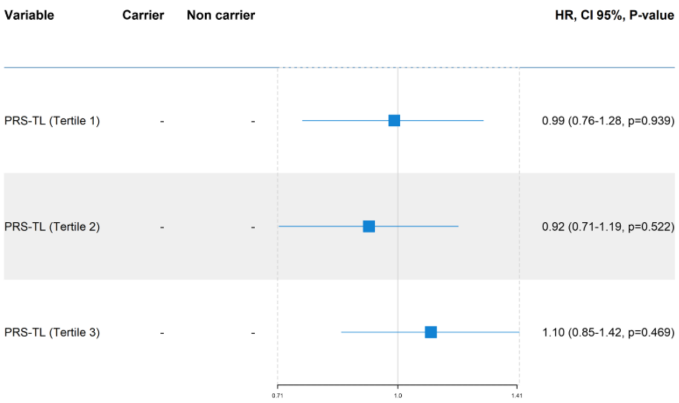

Supplementary Figure 12. Association between PRS-TL tertiles and survival in the PFFPR. A) Kaplan-Meier survival analysis for PRS-TL tertiles (p-value for the log-rank test is shown). B) PRS-TL effect on survival. The analysis was performed using Cox regression models adjusted for sex, age of diagnosis, the two main principal components, smoking history, forced vital capacity (FVC) % predicted, and diffusing capacity for carbon monoxide (DLCO) % predicted. The X-axis shows Hazard-ratios (HR); the grey solid line corresponds to the HR=1.0. The boxes correspond to adjusted HR and horizontal lines correspond to 95% confidence intervals (CI).

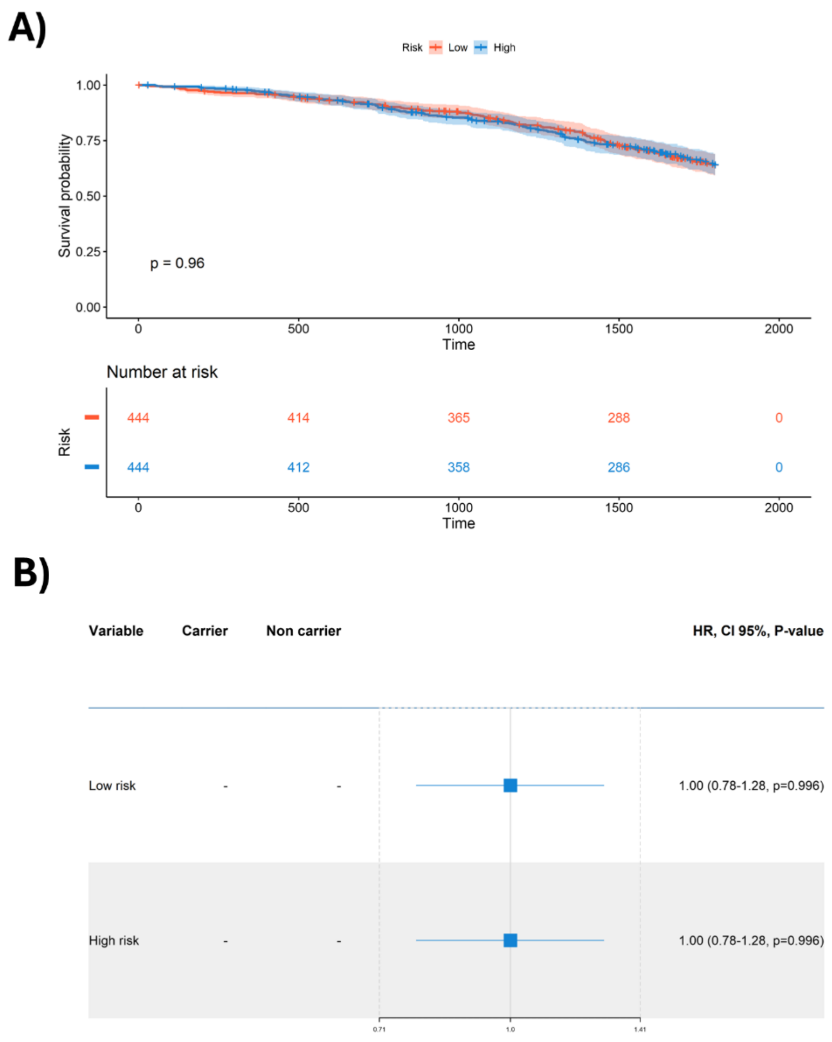

Supplementary Figure 13. Association between high and low PRS-TL and survival in the PFFPR. A) Kaplan-Meier survival analysis for high/low risk PRS-TL (p-value for the log-rank test is shown). B) PRS-TL effect on survival. The analysis was performed using Cox regression models adjusted for sex, age of diagnosis, the two main principal components, smoking history, forced vital capacity (FVC) % predicted, and diffusing capacity for carbon monoxide (DLCO) % predicted. The X-axis shows Hazard-ratios (HR); the grey solid line corresponds to the HR=1.0. The boxes correspond to adjusted HR and horizontal lines correspond to 95% confidence intervals (CI).

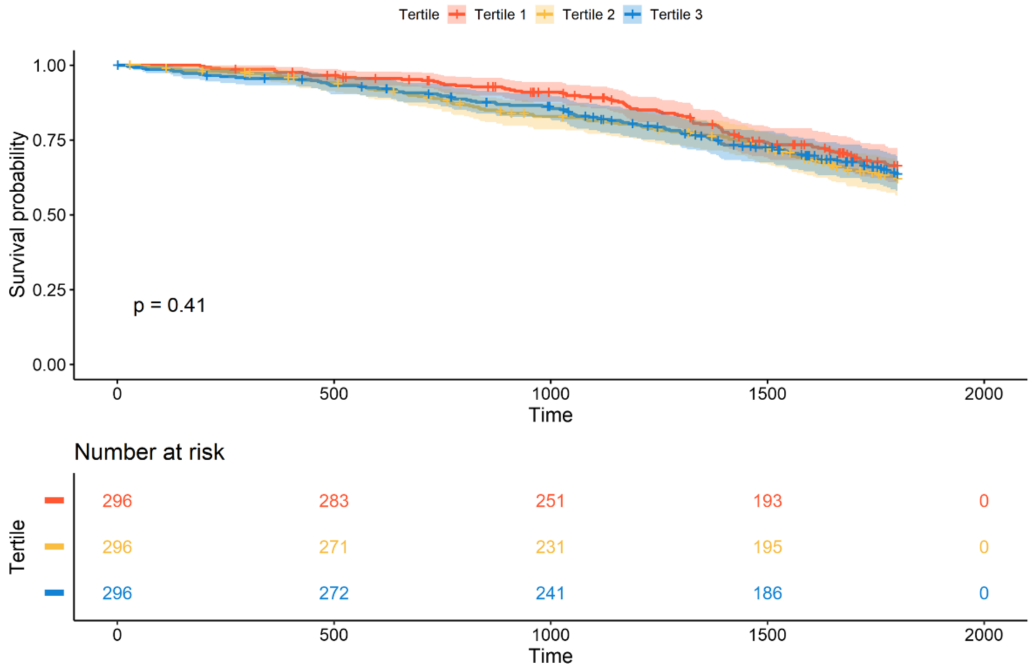

Supplementary Figure 14. Association of PRS-IPF (after excluding the *MUC5B* locus) and survival in the PFFPR. Kaplan-Meier analysis showing p-values for the log-rank test.

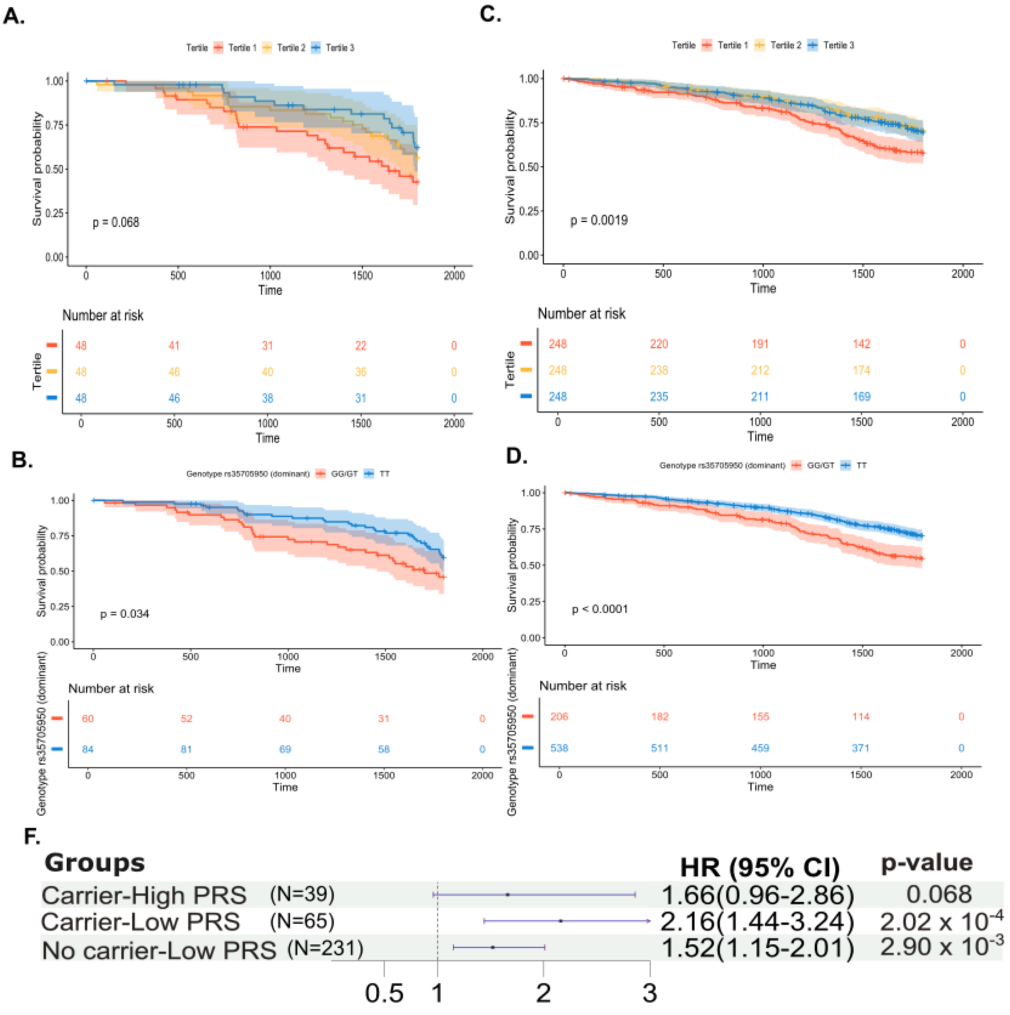

Supplementary Figure 15. Associations between PRS-IPF and *MUC5B* rs35705950 genotypes with survival among carriers and non-carriers of qualifying variants (QV) in the PFFPR. A) Association between PRS-IPF and survival in carriers. B) Association between PRS-IPF and survival in non-carriers. C) Association between *MUC5B* rs35705950 genotypes and survival in carriers. D) Association between *MUC5B* rs35705950 genotypes and survival in non-carriers. Kaplan-Meier analysis, showing p-values for the log-rank test. E) Cox regression analysis for subgroups of patients combining information of PRS-IPF and carrier status of QVs. The reference group was defined as non-carriers with high polygenic risk (N=257).

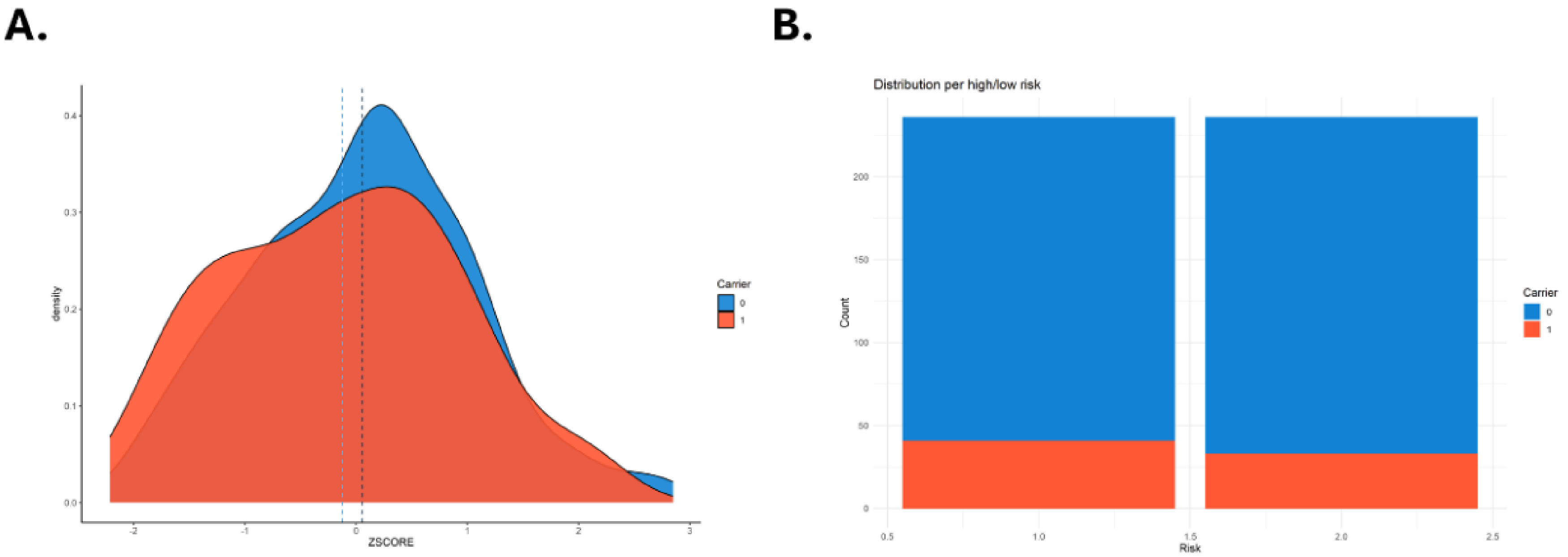

Supplementary Figure 16. Association between prevalence of qualifying variants (QV) and PRS-IPF in PROFILE. A) Distribution of PRS-IPF in carriers (1) and non-carriers (0). Vertical dotted lines represent the mean value of the distribution. B) Distribution of carriers (1) and non-carriers (0) in high and low PRS-IPF.

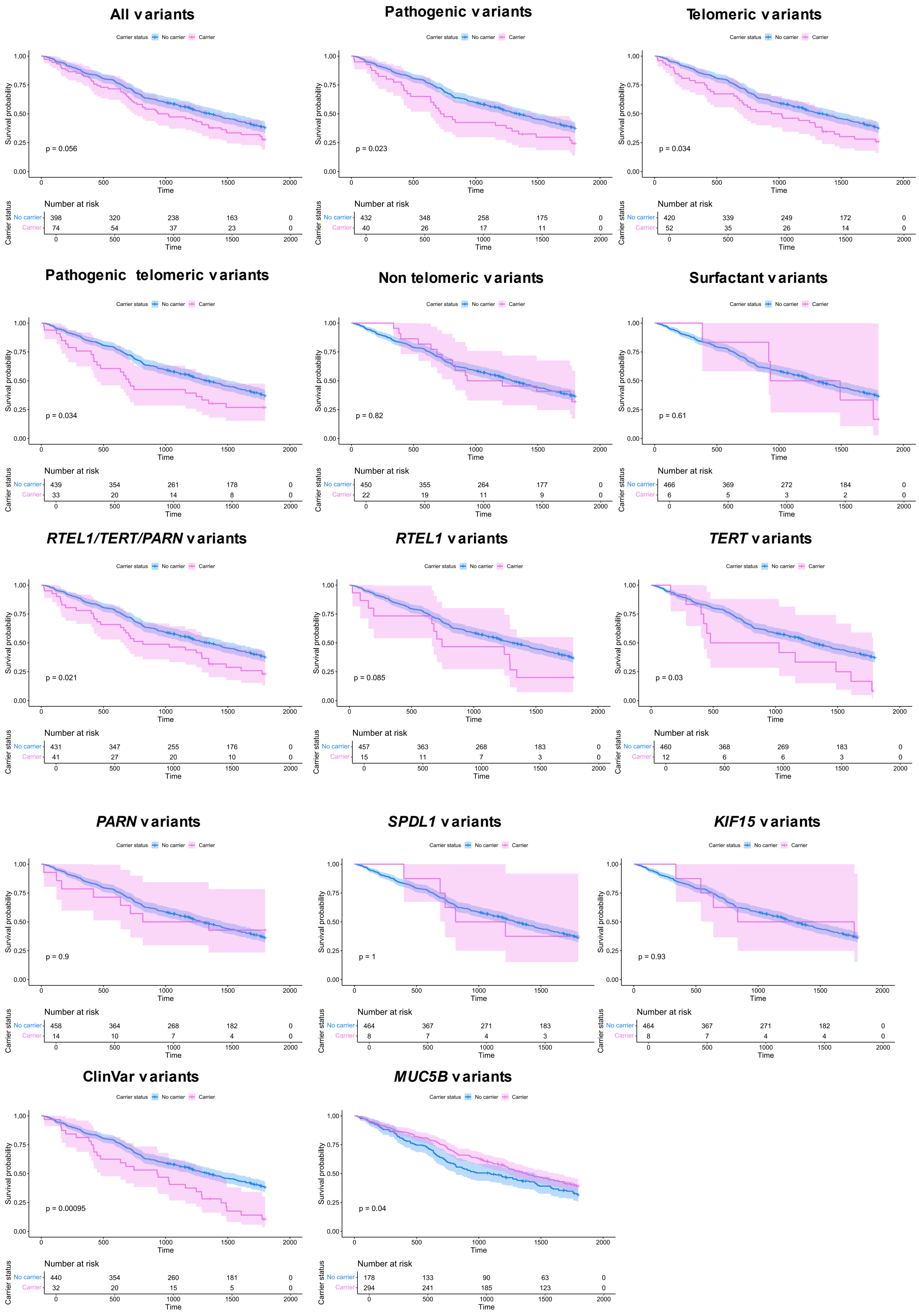

Supplementary Figure 17. **Kaplan-Meier survival analysis for qualifying variants (QV) (per gene and group PF genes) and the *MUC5B* risk allele in PROFILE. p-values for the log-rank test are shown.**

Supplementary references

1. Wang, B. R. *et al.* The Pulmonary Fibrosis Foundation Patient Registry. Rationale, Design, and Methods. *Ann Am Thorac Soc* **17**, 1620–1628 (2020).
2. Maher, T. M. PROFILEing idiopathic pulmonary fibrosis: rethinking biomarker discovery. *European Respiratory Review* **22**, 148–152 (2013).
3. Maher, T. M. *et al.* An epithelial biomarker signature for idiopathic pulmonary fibrosis: an analysis from the multicentre PROFILE cohort study. *The Lancet Respiratory Medicine* **5**, 946–955 (2017).
4. Ding, Z. *et al.* Estimating telomere length from whole genome sequence data. *Nucleic acids research* **42**, e75–e75 (2014).
5. Lee, M. *et al.* Comparative analysis of whole genome sequencing-based telomere length measurement techniques. *Methods* **114**, 4–15 (2017).
6. Dhindsa, R. S. *et al.* Identification of a missense variant in SPDL1 associated with idiopathic pulmonary fibrosis. *Commun Biol* **4**, 1–8 (2021).
7. Zhang, D. *et al.* Utility of whole genome sequencing in assessing risk and clinically relevant outcomes for pulmonary fibrosis. *European Respiratory Journal* **60**, (2022).
8. Zhang, D. *et al.* Rare and Common Variants in KIF15 Contribute to Genetic Risk of Idiopathic Pulmonary Fibrosis. *Am J Respir Crit Care Med* **206**, 56–69 (2022).
9. Hollmén, M. *et al.* KIF15 missense variant is associated with the early onset of idiopathic pulmonary fibrosis. *Respir Res* **24**, 240 (2023).
10. Harrison, P. W. *et al.* Ensembl 2024. *Nucleic Acids Research* **52**, D891–D899 (2024).
11. Allen, R. J. *et al.* wide association study across five cohorts identifies five novel loci associated with idiopathic pulmonary fibrosis. 1–5 (2022) doi:10.1136/thoraxjnl-2021-218577.
12. Li, C. *et al.* Genome-wide Association Analysis in Humans Links Nucleotide Metabolism to Leukocyte Telomere Length. *Am J Hum Genet* **106**, 389–404 (2020).
